# Potential framework for fully resourced of peach pits by multi-recycling approaches

**DOI:** 10.1038/s41598-025-97977-2

**Published:** 2025-09-30

**Authors:** Zanpei Zhang, Nyuk Ling Ma, Shen Ding, Jingjiang Qiu, Qiang Jiao, Yong Lai, Ruili Gu, Yuanyuan Chen, Xiao Wang, Mingwan Li, Yue Liu, Wanxi Peng, Dangquan Zhang

**Affiliations:** 1 College of Forestry, Henan Agricultural University, Zhengzhou, 450046 China; 2 Faculty of Science and Marine Environment, University Malaysia Terengganu, 21300 Kuala Terengganu, Malaysia; 3 Engineering Technology Research Center of Henan Province for MEMS Manufacturing and Applications, School of Mechanics and Safety Engineering, Zhengzhou University, Zhengzhou, 450001 China; 4 Henan Institute of Food and Salt Industry Inspection Technology, Zhengzhou, 450003 China

**Keywords:** Peach Pits, Nano-catalysis, Full resourcing, Biochar, Adsorption, Sustainability, Natural hazards

## Abstract

The yield of peaches worldwide is approximately 21 million tons annually, with peach pits representing 10% of the waste generated, and the disposal of this quantity of biowaste into landfill or combustion is a common practice that has the potential to cause a number of adverse environmental impacts. To explore the utilization of peach pit as a biomass source, nano-catalysis and high-efficiency extraction were used for efficient recycling management. The findings of DSC-TG and Py-GC-MS indicate that the though nano catalyst exerts a minimal impact on the weight loss rate throughout the pyrolysis process, it is potentially able to influence the composition of the pyrolysis product. UPLC/Q-TOF MS results suggest that peach pits were rich in bioactive components, which have potential applications in biomedicines, food additives and bioenergy industries. Concurrently, the peach pits could be efficiently pyrolyzed into high quality biochar (approximately 39% recovery) at 400 °C. Moreover, the produced biochar exhibits a higher capacity for adsorption of the heavy metal Cd when compared with As and Pb. Therefore, suggesting a potential circular economic model for peach production industry.

## Introduction

Peach (*Prunus persica*) is a small deciduous tree belonging to the genus *Prunus*, which is native to China with a history of cultivation more than 4000 years^1,2^. Peaches are regarded as one of the most popular fruits around the world, with an estimated consumption in nearly 100 regions. China is the leading producer of peaches, followed by the United States, Japan and Italy^3,4^. The high nutrient value and abundance sugar and organic acid content of peach sarcocarp make it a high demand, contributing to 21 million tons production worldwide^5,6^. Statistics from FAO (http://www.fao.org/faostat/zh/#data/QC) showed that the global production of peaches was approximately 24.45 million tons from the year 2018 (Table S1) and this yield approximately 489,000 tons of peach pits (PPs). If this PPs is directly discarded onto landfill, the circulation pressure on the biosphere will be increased dramatically. Nevertheless, the waste management for food industry remains inefficient, with non-edible parts still being discarded to landfill in large quantities^7^. Due to the hard structure of PPs, it required long time for degradation, therefore accumulation of peach residue in back yard of peach fruit production site always release bad odor^8^. The high content of amygdalin in unripe peach may present a biological risk when disposed to landfill, as it can cause acute intoxication and chronic nervous problems^9^. Other management such as incineration also caused serious environmental effects including CO_2_ emission and generation of particulate materials and polycyclic aromatic hydrocarbons (HAP)^10^. Nevertheless, the high calorific value of PPs is suitable for biofuel production^8^ such as phenol, benzyl nitrile, 2-pentanone, and some other potential bioenergy sources. However, the biofuel production using peach pits require extra process as the rigid physical structure of peach does not easily deform which also increases the production cost.

It has been reported that recycling PPs have removal capacity of pesticide, pollutant and hexavalent chromium^2,11,12^. Thermal cracking is a process by which biomass could be treated at a high temperature without oxygen to obtain the biochar^13–15^. Biochar has been demonstrated to enhance the soil environment and adsorb hazardous substances during food processing^16–19^. Therefore, this study aims to explore the potential of complete resourcing utilization of PPs. The bioactive ingredients in PPs were extracted by solid–liquid extraction technology and then analyzed using Fourier transform infrared spectrometer (FTIR) and ultra-high performance liquid chromatography-quadrupole time-of-flight mass spectrometry (UPLC/Q-TOF MS) technologies. Waste conversion techniques, including simultaneous thermal analyzer (STA), nano-catalysis technology and pyrolysis technology, were combined to produce biochar. Furthermore, morphological characterization and adsorption experiments were conducted out on the PPs biochar.

## Materials and methods

### Materials preparation

Following the removal of kernels, the PPs were air-dried at room temperature (25 ~ 30 °C) pulverized. Thereafter, the sample powder was sieved through a 200-mesh sieve and subsequently stored under dry and ventilated conditions.

### Extraction of active compounds

The sample powders were extracted with ethanol, benzene, or acetone at the ratio of solid–liquid 1: 20, respectively. The samples were immersed for eight hours at room temperature, and then completely extracted with an automatic Soxhlet extraction apparatus in ethanol, benzene and acetone at 79 °C, 60 °C, and 56 °C for 4 h. After the removal of the extracted residues, the filtrate was evaporated at 40 °C under a vacuum rotary evaporator until concentrated to 10 mL, and the extracted residues were dried and stored at 25 °C.

### FTIR and UPLC/Q-TOF MS analysis

After the sample was sufficiently dried, 1 mg of powder was added with 100 mg of potassium bromide and mixed well to form a tablet. This tablet is placed under an infrared lamp and dried before proceed with FTIR analysis (Nicolet IS 5) from 4000 cm^−1^ to 500 cm^−1^. For UPLC/Q-TOF MS (Agilent 1290 UPLC/ Agilent 6540 Q-TOF) analysis, sample was passed through column ACQUITY UPLC® BEH C18 1.7 μm × 2.1 mm × 50 mm with setting capillary, desolvation temperature is 350 °C and desolvation gas is 800 L·h^−1^, mass spectrometry ESI + sensitivity was about 100 ~ 1200 Pa, ramp collision energy is between 10 and 40 V, and cone voltage is 25 V.

### Preparation of PPs biochar

PPs powder (15 g) was placed into the hot kiln with a quartz boat and burned in a nitrogen atmosphere. The temperature in the hot kiln was increased to 400 °C or 600 °C at the rate of 10 °C·min^−1^ and hold for 2 h. Upon the completion of the heating process, the quartz boat is permitted to cool down to 35 °C and the yield of biochar is then calculated using the following formula:

1 $${\text{M}} = \, \left( {{\text{m}}_{{1}} /{\text{m}}_{0} } \right) \, \times { 1}00\%$$

M means the yield of PPs biochar (%), m_1_ represents the weight of PPs biochar (g), m_0_ is the weight of PPs powder (g).

The morphology of the biochar was characterized by scanning electron microscope (SEM, Zeiss Gemini 300).

### Adsorption capacity of PPs biochar

#### Adsorption capacity for heavy metals

The initial ion concentration exerted a significant influence on the adsorption capacity of biochar. When the metal ion concentration was within the range of 10 ~ 100 μg·mL^−1^, the ion adsorption capacity of biochar exhibited a pronounced increase with the rise in concentration (until the ion concentration reached 50 μg·mL^−1^), after which it gradually tended to stabilize or exhibit a slight decrease. According to the previous studies^11,13^, arsenic (As), lead (Pb) and cadmium (Cd) at concentrations of 10 μg·mL^−1^, 50 μg·mL^−1^ and 100 μg·mL^−1^ have been prepared into 20 mL. An amount of PPs biochar 0.04 g was added into the 20 mL heavy metal solution and allowed adsorption to occur for 60 min at 25 °C, pH =7, and 200 rpm. The PPs biochar was removed by filtration and the remaining solution was examined by Inductively Coupled Plasma Mass Spectrometer (ICP-MS, Agilent 7900). The formula for calculating the adsorption capacity of PPs biochar for heavy metals is as follow:

2 $$\text{Q }=\frac{\text{(C-}{\text{C}}_{0}\text{)* V}}{\text{m}}$$

Q (mg·g^−1^) represents the adsorption capacity of PPs biochar to heavy metals, C (mg·mL^−1^) means the concentration of heavy metals after adsorption and C_0_ (mg·mL^−1^) is concentration of heavy metals before adsorption, the total volume of heavy metal solution is V (mL) and m (g) means the weight of PPs biochar.

#### Adsorption capacity for methylene blue

A range of PPs biochar at concentration 1 g·L^−1^, 2 g·L^−1^ and 4 g·L^−1^ was added to 20 mg·L^−1^ of methylene blue solution separately. The reaction is allowed to take place at 28 °C, 200 rpm, pH = 7 for 60 min. After 60 min, the PPs biochar was removed and the remaining solution was analyzed at 664 nm using UV spectrophotometer (UV-9000S).

### Effect of nano catalysts on biochar production

The effect of nano-catalysts on the pyrolysis process of PPs samples were tested by adding catalysts nano-Mo, nano-Co_3_O_4_ or nano-Mo/Co_3_O_4_ to the samples at the ratio of 1:100, and the pyrolysis effect were analyzed by using differential scanning calorimeter-thermogravimetric analyzer (DSC-TG, NETZSCH STA 449C) and Pyrolysis Gas Chromatography-Mass Spectrometry (Py-GC-MS). For thermal analyze, dried peach powder with catalysts (10 mg) was injected into DSC-TG analyzer. The temperature was set from 50 to 800 °C with a heating rate of 10 °C·min^−1^. The degradation of PPs with catalysts under inlet conditions is tested by Py-GC-MS. Dried peach powder with catalysts (10 mg) was injected in a pyrolysis tube (CDS, USA) and the tube was sealed with glass wool and placed in a cracker injector (CDS 6250 T). The pyrolysis process was operated at an initial temperature of 50 °C for one second, then increased to 550 °C or 700 °C, and held for 10 s. The product during pyrolysis was analyzed using GC-MS (Agilent 7890B-5977) using HP-5 capillary column 30 m (length) × 0.25 mm (inside diameter) × 0.25 μm (film thickness). All data were processed by Agilent Chem Station and Excel 2010 software.

## Results and discussion

### Functional group changes of PPs

FTIR is a rapid detection technology with high sensitivity that can quickly and efficiently identify chemical bonds and functional groups of many types of compounds^20^, consequently, it is widely used for preliminary determination of substances^21,22^. The extract and the original solid powder were analyzed by FTIR and compared in order to reveal the change in the chemical groups when extract by different extraction method. It can be seen from the infrared curve that there are 7 peaks that arise in the original powder of PPs, while 7, 4, and 3 peaks are observed in the ethanol extract, acetone extract, and benzene extract respectively. Furthermore, the infrared spectrum curve of the ethanol extract exhibited a similar trend to that of the original powder of PPs, indicating that ethanol is the most effective solvent for extracting the ingredients in PPs.

**Fig. 1:**
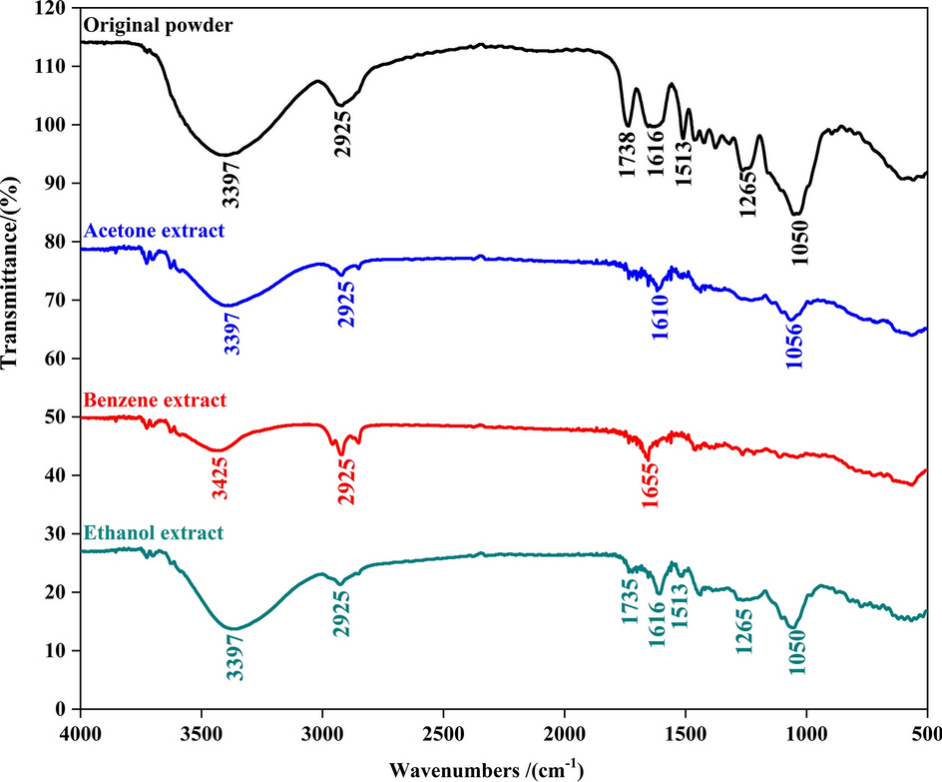
The FTIR spectra of the original powder and samples extracted by acetone, benzene and ethanol solution.

In the fully functional group region, there was an absorbance peak corresponding to the free hydroxyl stretching vibration at 3397 cm^−1^, which was most probably attributable to phenolic compounds were present in the raw PPs sample (Fig. 1). Additionally, absorption peaks were observed at 2925 cm^−1^, 1738 cm^−1^, 1616 cm^−1^, 1513 cm^−1^, 1265 cm^−1^ and 1050 cm^−1^, indicating the stretching and oscillating vibration of the hydrogen-oxygen bond, carbon-carbon double bond, carbon-oxygen bond and alcoholic compounds in PPs^23,24^. The results indicates that the alcohols, ketones, phenols, aldehydes, acids, and even olefins compounds in the PPs can be extracted by ethanol solution. Therefore, the ethanol solution could be selected as extracting agent.

### Non-volatile components in extracts of PPs

**Fig. 2:**
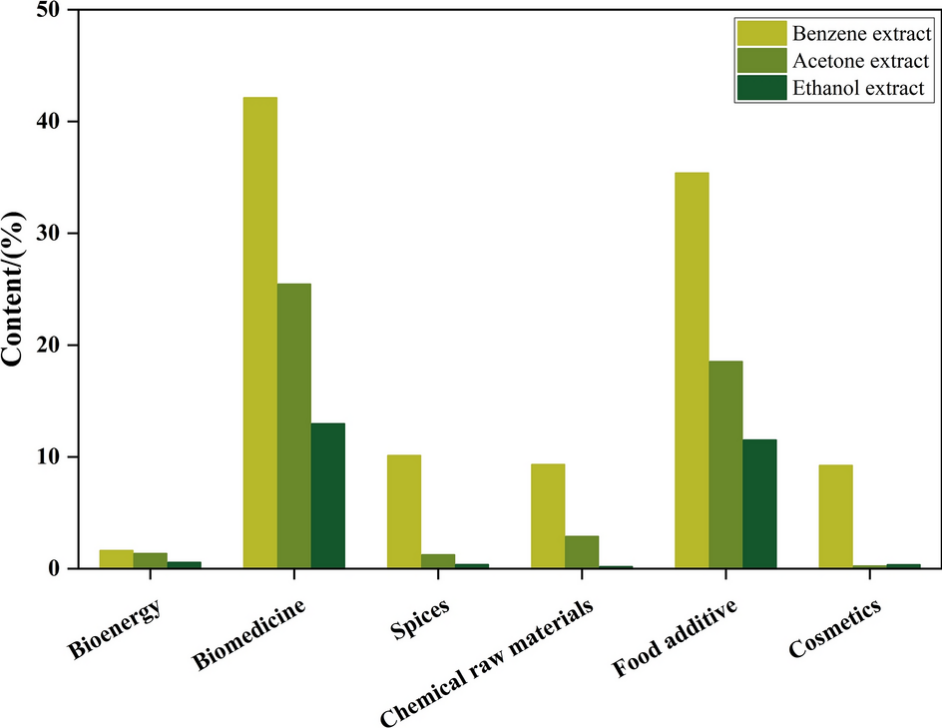
The classification of functional components in the three extracts.

The UPLC/Q-TOF MS identified 171 and 164 compounds in ethanol and acetone extractions but only 77 substances in benzene extractions (Supplementary Figure S1 to S3, Supplementary Table S2 to S4). These compounds can be further categorized into bio-medicine, bio-energy, spices, cosmetics and food additives according to their main functions (Fig. 2, Supplementary Table S5 to S7). The extraction of substances from PPs varies according to polarities of the different solvents employed, as a consequence of differing solvent polarity. The same ingredients were observed in different extractions, including 1-methyl-6, 7-dihydroxy-3, 4-dihydroisoquninoline monohydrate, 3-methoxy-4-hydroxybenzoic acid, 2-methoxycinnamic acid, pulmonene, glycyrrhizin, octadecedenoic acid and cimifugin, which mainly used as bio-medicine^25,26^ while the m-methoxycinnamic acid could be used in the field of cosmetic or spices^27^.

### The yield of PPs biochar

The yield of PPs biochar is 38.92% at 400 °C and the yield of PPs biochar at 600 °C is 28.07% (Table 1), indicating that the yield of biochar at 400 °C is approximately 11% higher than at 600 °C.

**Table 1:** The PPs biochar yield at different temperatures.

No	T/°C	Biochar yield /%
1	400	38.92 ± 0.20b
2	600	28.07 ± 0.27a

Different letters indicate that the biochar yield is significant at different temperatures (*P* < 0.05).

**Fig. 3:**
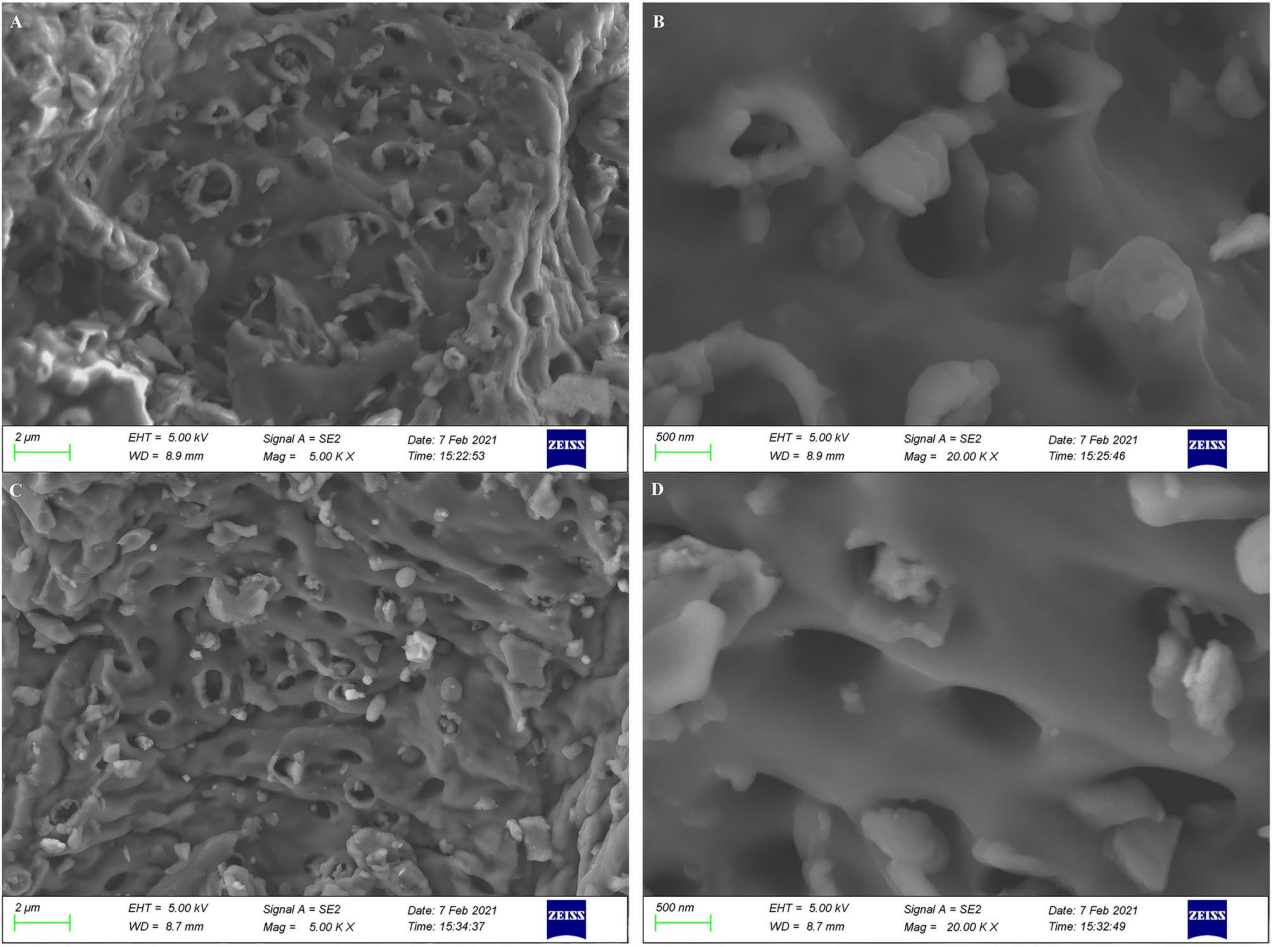
The morphology of the PPs biochar observed by SEM. (**A**) & (**B**) are biochar prepared at 400 °C observed at magnification 5000 & 20,000 respectively; (**C**) & (**D**) are biochar prepared at 600 °C and observed at magnification 5000 & 20,000 respectively.

The SEM image of the PPs biochar (Fig. 3) reveals that the surface of the PPs biochar prepared at 400 °C and 600 °C is not smooth, accompanied by wrinkles and holes (Fig. 3A and C), and the pores size of the PPs biochar is 500 nm (Fig. 3B and D). The results of SEM demonstrated that there was no significant difference in the pore size of biochar obtained by two different temperatures. In consideration of both the yield and morphological characteristics of biochar, the biochar prepared at 400 °C was selected as the adsorbent for the subsequent adsorption experiment.

### Heavy metal and methylene blue removal

**Fig. 4:**
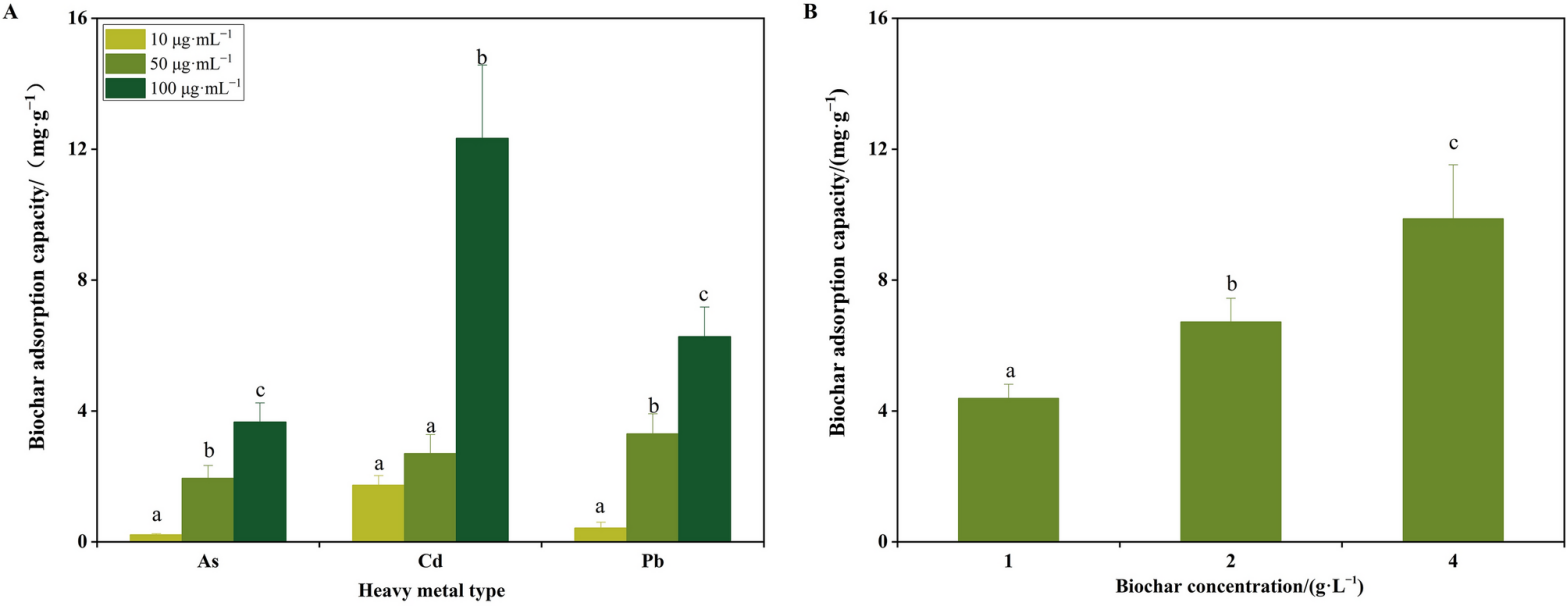
The adsorption capacity of PPs biochar for heavy metals (**A**) and methylene blue (**B**). (**A**) Different letters indicate that the adsorption capacity of biochar is significant between different concentrations of the same heavy metal (*P* < 0.05); (**B**) The adsorption capacity of methylene blue in different concentration of PPs biochar. Different letters indicate that there is a significant difference in the removal rate of methylene blue by the amount of biochar added (*P* < 0.05).

The heavy metal removal for As at concentrations of 10 μg·mL^−1^, 50 μg·mL^−1^, and 100 μg·mL^−1^ is 8.53 μg, 77.45 μg and 146.35 μg, respectively. The heavy metal removal of Cd is 69.10 μg, 107.77 μg and 493.24 μg, while the Pb removal is 16.88 μg, 132.02 μg and 250.90 μg in three concentrations. The adsorption results demonstrated that PPs biochar exhibits the capacity to remove As, Cd and Pb. Furthermore, the adsorption capacity of PPs biochar exhibits a notable increase as the concentration of the adsorbate increases (Fig. 4A). Comparing the adsorption capacity of PPs biochar and heavy metal removal to As, Cd and Pb, it can be concluded that whether it is a low-concentration or high-concentration solution, the heavy metal removal of PPs biochar has the best adsorption effect on Cd.

The results of the UV spectrophotometer showed that the PPs biochar was capable of reducing the concentration of methylene blue in the water (Fig. 4B). The degradations of methylene blue were 4.38%, 6.72% and 9.87%, respectively. It was observed that the removal ability significantly increased as the amount of biochar used increased. However, the adsorption capacity of methylene blue per gram of biochar was found to be reduced. Environmental pollution activities caused by human behaviour have attracted global attention^28^, harmful substances produced by factories such as printing factories, leather factories and textile factories could result to deterioration of our environment^29–31^. The heavy metals easily penetrate into the soil and the long-term accumulation in the soil will threaten human health^32,33^.

It has been well demonstrated that biochar can adsorb heavy metals in the soil, thereby reducing contaminant bioavailability in the field^34–36^. Furthermore, biochar can bind soil nutrient elements and releases them gradually back into soil solution^37^, retain water to decrease evaporation and improve soil structure, thus providing a more conducive environment for plant growth^38–40^. Conversion of PPs and recycled into biochar is a promising approach for reducing the waste burden in landfill. Moreover, biochar has the potential to be utilized for heavy metal removal in water and soil^41,42^, which could significantly reduce the hazard to human health^43^. Additionally, biochar may have potential applications in contaminant remediation, whereby it can act as a sorbent for organic and inorganic contaminants^44,45^.

### Nanomaterials and pyrolysis process

**Fig. 5:**
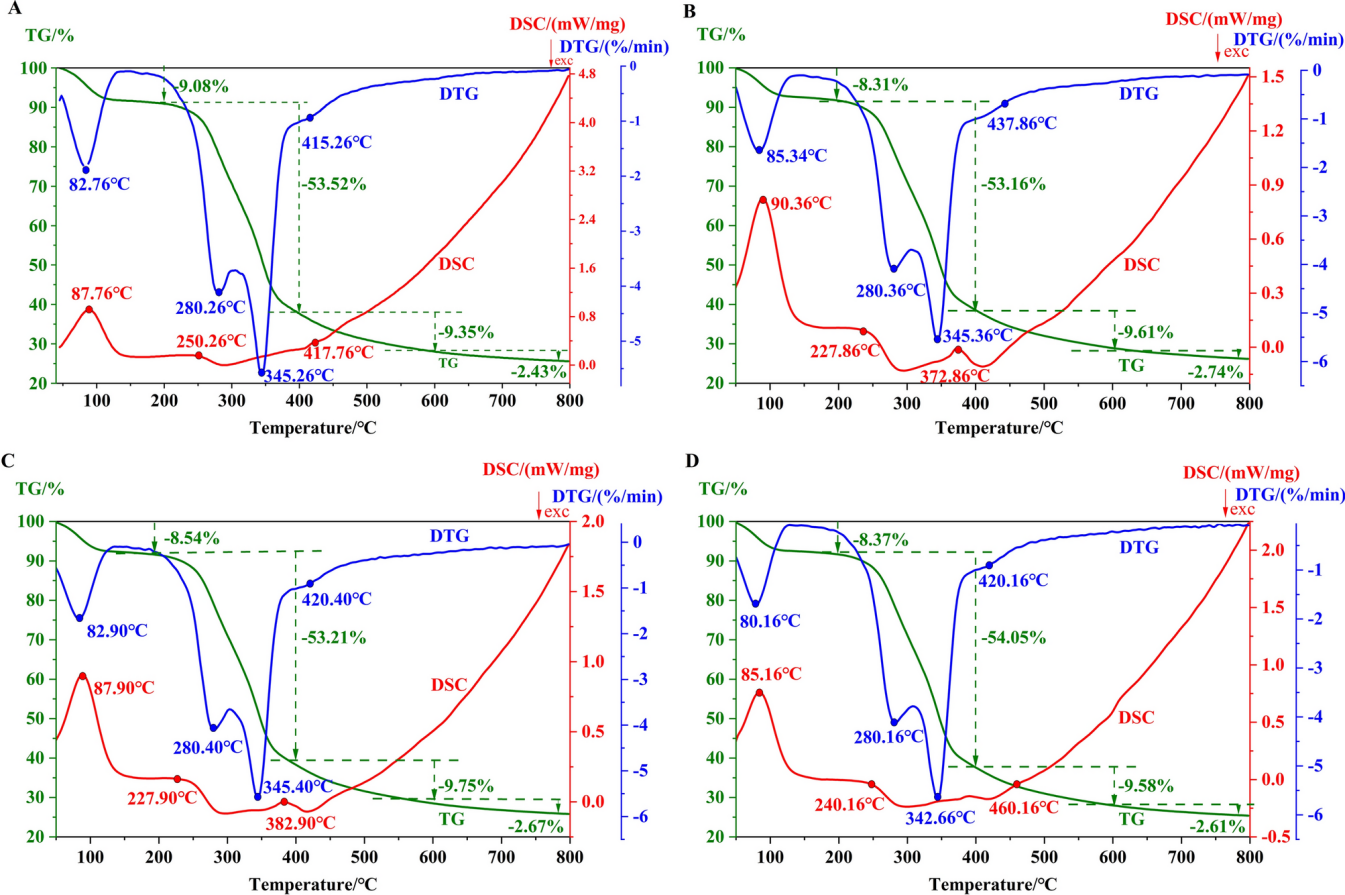
The DSC-TG-DTG curves of the PPs samples. (**A**) Raw PPs samples without catalyst; (**B**) PPs-Mo samples; (**C**) PPs-Co_3_O_4_ samples; (**D**) PPs-Mo/ Co_3_O_4_ samples.

To explore and maximize the usage and increase the production of biochar, nanomaterials were used in pyrolysis process (Fig. 5). The nano-catalyst and the original powder of PPs were mixed and detected with DSC-TG analyzer. The DTG curve shows three extreme values during the pyrolysis process at 85 ± 3 °C, 280 ± 1 °C, and 345 ± 3 °C. The temperature point and the weight loss rate indicated the pyrolysis process of the sample in the TG curve can be divided into four stages, including 45 ~ 200 °C, 200 ~ 400 °C, 400 ~ 600 °C, and 600 ~ 800 °C. The DSC curve reveals that the nano catalyst has a negligible effect on the enthalpy change during the pyrolysis process, but it may have an effect on the components in the product after pyrolysis^46,47^. The maximum weight loss approximately 54% is observed in the range of 200 ~ 400 °C when water and degradation mainly occur at this point. The biochar was obtained at a higher temperature around 600 ~ 800 °C where a more stable weight loss of approximately 3% was observed.

**Fig. 6:**
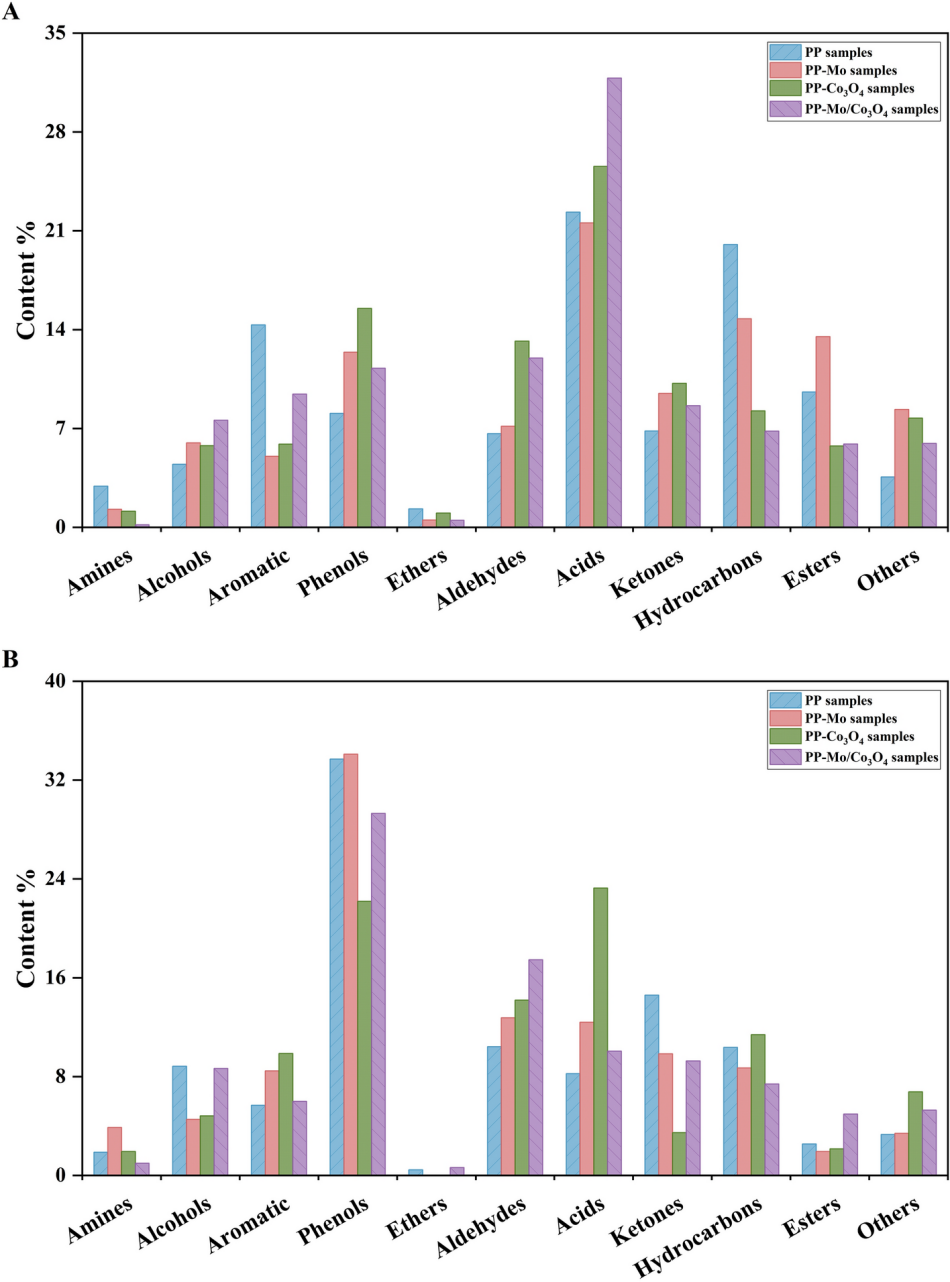
The classification of substances obtained during pyrolysis of non-catalytic PPs samples, the PPs-Mo samples, PPs-Co_3_O_4_ samples and PPs-Mo/Co_3_O_4_ samples at (**A**) 550 °C and (**B**) 700 °C.

Rapid pyrolysis technology under inlet oxygen condition effectively convert biomass into functional products such as bio-gas and bio-oil. However, both of bio-gas and bio-oil exhibit complicated compositions and different ratios of chemical compounds^48–50^. The application of nano-catalysis technology may improve the proportions of the complicated compositions^51–53^. To explore the potential for multi-stage utilization of PPs, the nano-catalysis technology combined with pyrolysis and the products during pyrolysis were further investigated. According to the results of DSC-TG, 550 °C and 700 °C were selected as the temperature for exploration. For all of the PPs samples, the total ion chromatograms were shown in the Supplementary Fig. S4 to S11, and the compositions were shown in the Supplementary Table S8 to S15, the composition of the PPs samples were classified by common functional groups (Fig. 6). The nano-catalytic and non-catalytic PPs samples at 550 °C (Fig. 6A) and 700 °C (Fig. 6B) during pyrolysis contain the same products, but the functional proportions were different. The content of acids was the highest at 550 °C, while at 700 °C, the content of phenols was reached its maximum.

Py-GC-MS results showed that adding catalyst retrieved more chemical substances (more than 100) compared to the raw sample without catalyst. Furthermore, the temperature of 700 °C allows for the formation of a more diverse range compared to 550 °C. Py-GC/MS analysis indicated that the non-catalytic PPs samples at 700 °C were more efficient at producing alcohols, phenols and ketones, while aromatic, acids and esters are more abundance at 550 °C. The substances acetic acid, furfural, phenol, 2-methoxy-, creosol, 2-methoxy-4-vinylphenol, 4-(1E)-3-Hydroxy-1-propenyl-2-methoxyphenol and n-hexadecanoic acid were observed, and they can be used in the field of biomedicine, bioenergy, food additives and chemical materials^54–56^. As an example, n-hexadecanoic acid can be used as a medium for storing energy, which reduced the energy consumption and maintained the ecological balance and ecological environment^57,58^. Furfural is a raw material for the preparation of many drugs and industrial products, which can be used to prepare oxalic acid, furfuryl alcohol, preservatives, etc^59,60^. Interestingly, the utilization of nano-Mo/Co_3_O_4_ at 700 °C could increase the contents of aldehydes about 5% ~ 11%. Aldehydes can be oxidized into acids or esters and are widely used in industry fragrance^61^.

**Fig. 7:**
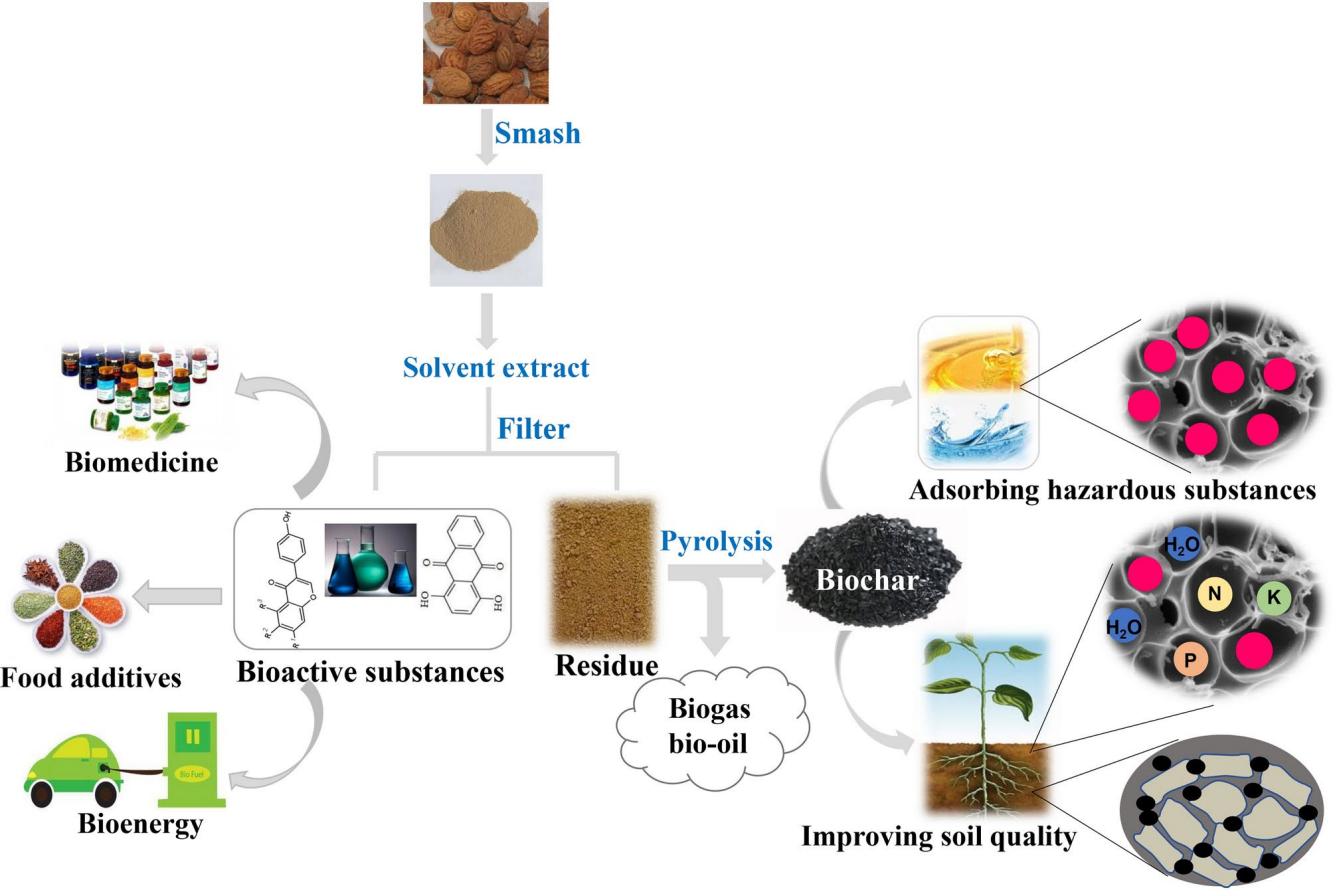
A multi-stage utilization model for wasted PPs. Bioactive substances could be obtained by various extraction process, where it can be downstream application for biomedicines, food additives and bioenergy industries. The residue after extraction can be pyrolysed to produce biogas, bio-oil and biochar. Biochar can be applied for soil and water improvement (eg hazardous substances removal).

According to previous studies, biochar is a common adsorbent^62,63^, the modified biochar could improve its absorption ability^64–66^. However, in our observation, the addition of catalyst does not significantly increase the biomass conversion of biochar (Fig. 5) and the chemical constitution in biochar without catalyst also shows a similar profile as PPs biochar, therefore it is not economically feasible to add catalyst into biochar production. The DSC-TG results revealed that the conversion rate of PPs biochar was at least 26% at 800 °C, which means the annual production of PPs biochar could reach approximately 127,100 tons if this approach were employed. Moreover, the chemical content of PPs biochar observed in Py-GC-MS provide a broad prospect of biochar produced from biomass waste in for agriculture and pollution abatement applications (Fig. 7). Of course, in addition to biochar, biomass waste can also be used to produce fuels and chemical feedstocks through the pyrolysis process^67,68^.

## Conclusions

PPs contain phenols, ketones, acids and alcohols, which can be recycled through extraction and pyrolysis techniques. Biochar produced under nitrogen atmosphere is of high quality and can effectively remove heavy metals, including As, Pb, and Cd, in concentrations ranging from 10 to 100 mg·L^−1^. Furthermore, the PPs biochar has the capacity to remove methylene blue dye. The proposed framework in this research demonstrates a circular management of PPs waste for multiple applications. The findings of this study provided a theoretical foundation for the optimal utilisation of PPs, in particular for PPs biochar in various applications.

## Supplementary Information

Supplementary Information.

## Data Availability

The data supporting the findings of this study are available within the paper and supplementary information.
