# Supplementary material for: Potential framework for fully resourced of peach pits by multi-recycling approaches
Source: Sci Rep. 2025 Sep 30;15:33761. doi: 10.1038/s41598-025-97977-2 (PMC12484624; doi:10.1038/s41598-025-97977-2)
Supplement: Supplementary file 1 — Supplementary Information. [file 41598_2025_97977_MOESM1_ESM.pdf]

Supplementary Information

Potential Framework for Fully Resourced of Peach Pits by Multi-Recycling Approaches

Figures

Figure S1 UPLC/Q-TOF MS total ion chromatograms of benzene extract from PPs samples

Figure S2 UPLC/Q-TOF MS total ion chromatograms of acetone extract from PPs samples

Figure S3 UPLC/Q-TOF MS total ion chromatograms of ethanol extract from PPs samples

Figure S4 Py-GC-MS total ion chromatograms of non-catalytic PPs samples at 550 ℃

Figure S5 Py-GC-MS total ion chromatograms of PPs-Mo samples at 550 ℃

Figure S6 Py-GC-MS total ion chromatograms of PPs-Co_3O_4 samples at 550 ℃

Figure S7 Py-GC-MS total ion chromatograms of PPs-Mo/Co_3O_4 (1:1) samples at 550 ℃

Figure S8 Py-GC-MS total ion chromatograms of non- catalytic PPs samples at 700 ℃

Figure S9 Py-GC-MS total ion chromatograms of PPs-Mo samples at 700 ℃

Figure S10 Py-GC-MS total ion chromatograms of PPs-Co_3O_4 samples at 700 ℃

Figure S11 Py-GC-MS total ion chromatograms of PPs -Mo/ Co_3O_4 (1:1) samples at 700 ℃

Tables

Table S1 The distribution area and production of peaches

Table S2 UPLC/Q-TOF MS compositions of benzene extract from PPs samples

Table S3 UPLC/Q-TOF MS compositions of acetone extract from PPs samples

Table S4 UPLC/Q-TOF MS compositions of ethanol extract from PPs samples

Table S5 Classification of the UPLC/Q-TOF MS functional components in benzene extract

Table S6 Classification of the UPLC/Q-TOF MS functional components in acetone extract

Table S7 Classification of the UPLC/Q-TOF MS functional components in ethanol extract

Table S8 Py-GC-MS components of non-catalytic PPs samples at 550 ℃

Table S9 Py-GC-MS components of PPs-Mo samples at 550 ℃

Table S10 Py-GC-MS components of PPs-Co_3O_4 samples at 550 ℃

Table S11 Py-GC-MS components of PPs-Mo/Co_3O_4 (1:1) samples at 550 ℃

Table S12 Py-GC-MS components of non-catalytic PPs samples at 700 ℃

Table S13 Py-GC-MS components of PPs-Co_3O_4 samples at 700 ℃

Table S14 Py-GC-MS components of PPs-Co_3O_4 samples at 700 ℃

Table S15 Py-GC-MS components of PPs-Mo/ Co_3O_4 (1:1) samples at 700 ℃

Supplementary Figures

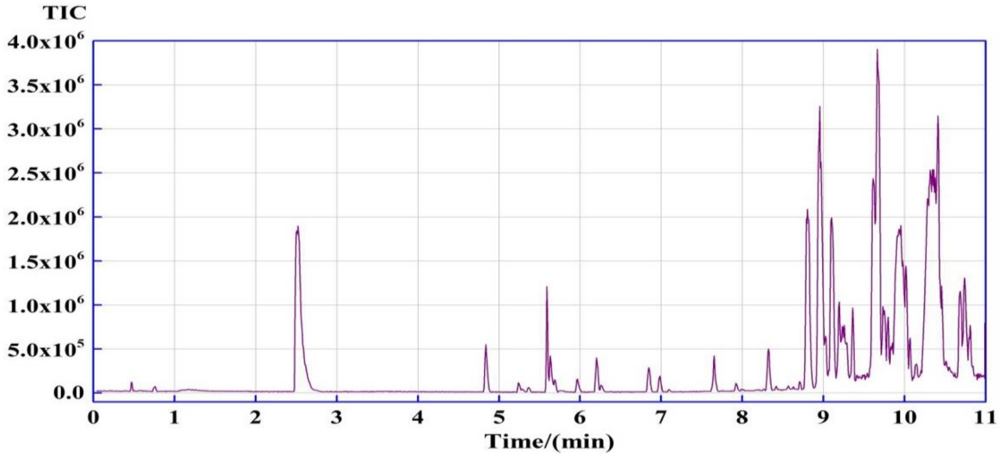

Figure S1 UPLC/Q-TOF MS total ion chromatograms of benzene extract from PPs samples.

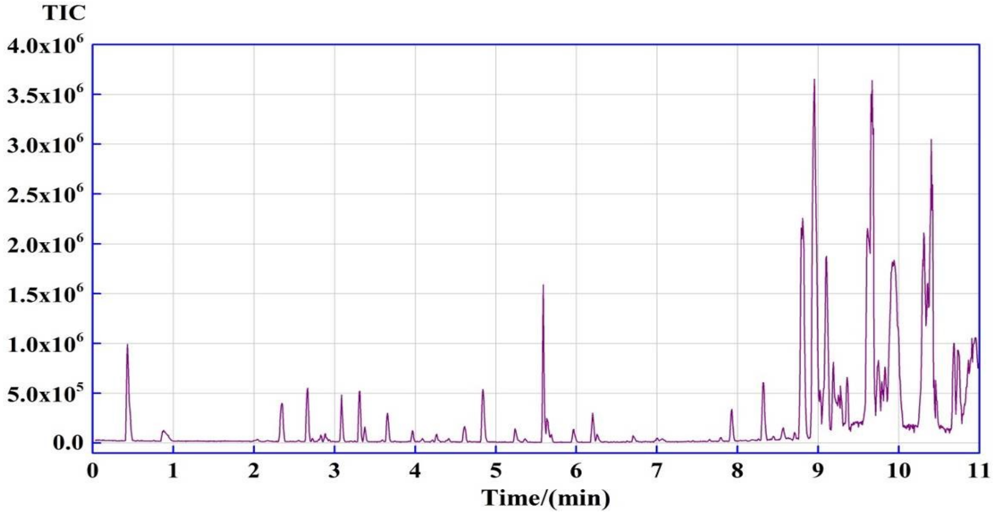

Figure S2 UPLC/Q-TOF MS total ion chromatograms of acetone extract from PPs samples.

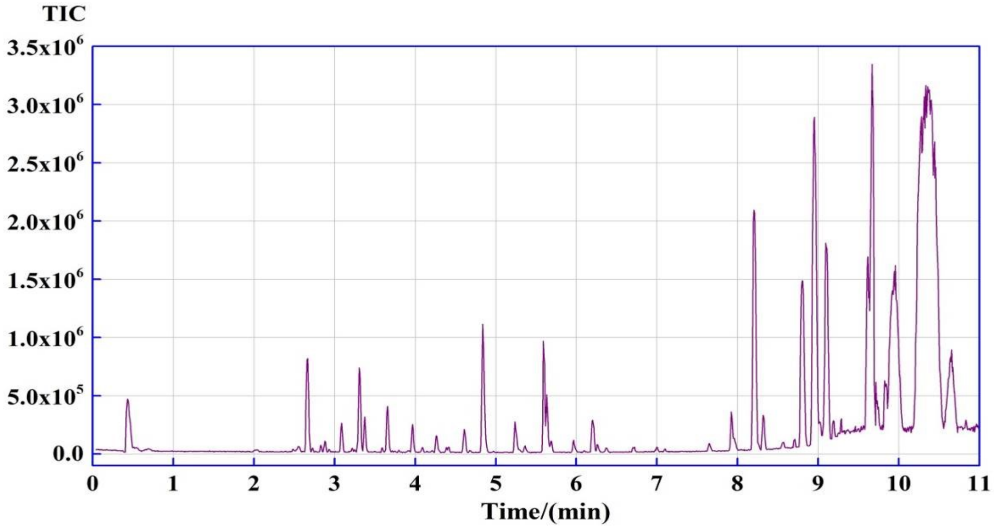

Figure S3 UPLC/Q-TOF MS total ion chromatograms of ethanol extract from PPs samples.

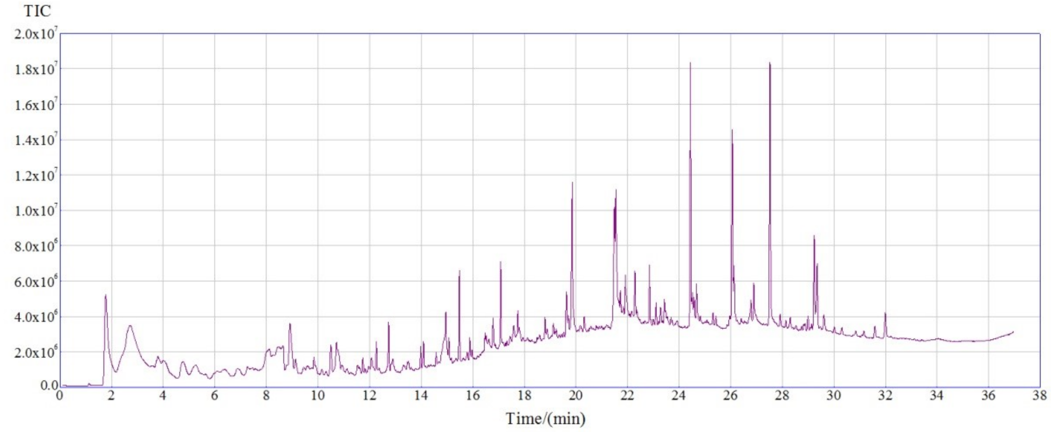

Figure S4 Py-GC-MS total ion chromatograms of non- catalytic PPs samples at 550 °C.

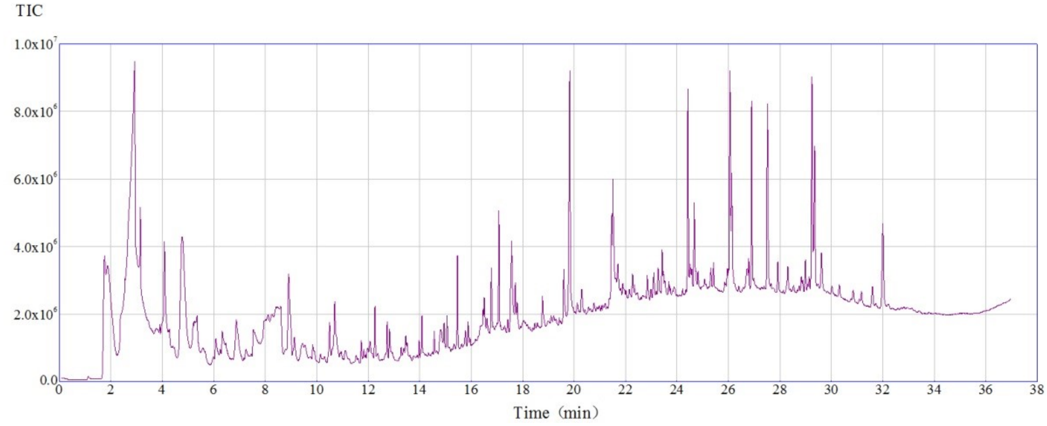

Figure S5 Py-GC-MS total ion chromatograms of PPs -Mo samples at 550 °C.

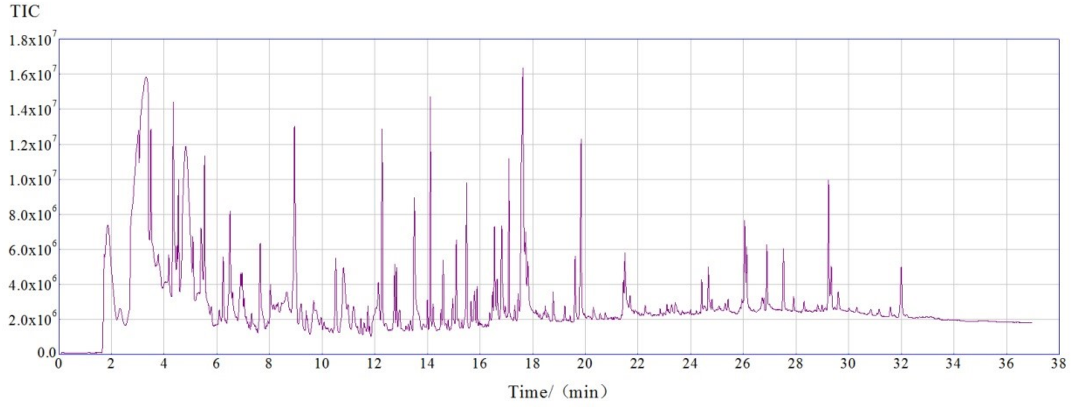

Figure S6 Py-GC-MS total ion chromatograms of PPs -Co3O4 samples at 550 °C.

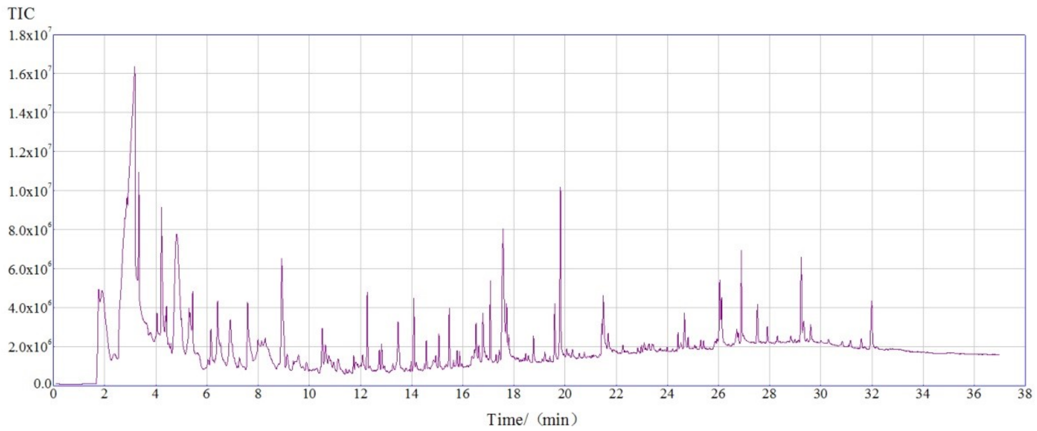

Figure S7. Py-GC-MS total ion chromatograms of PPs -Mo/Co3O4 (1:1) samples at 550 °C.

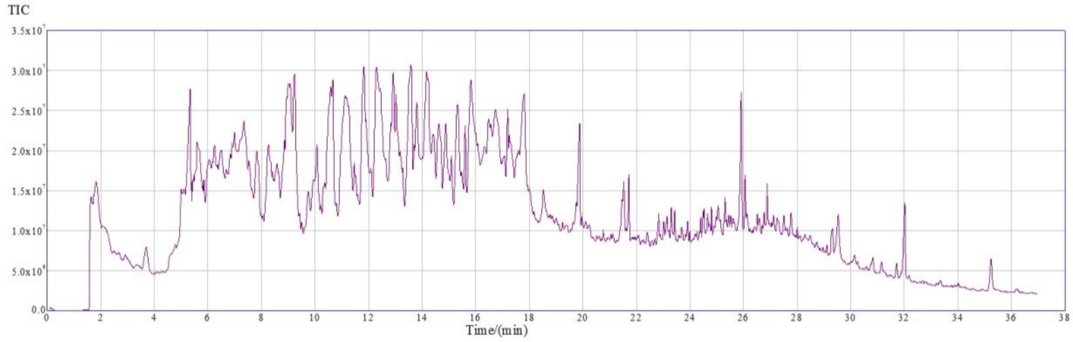

Figure S8. Py-GC-MS total ion chromatograms of non- catalytic PPs samples at 700 °C.

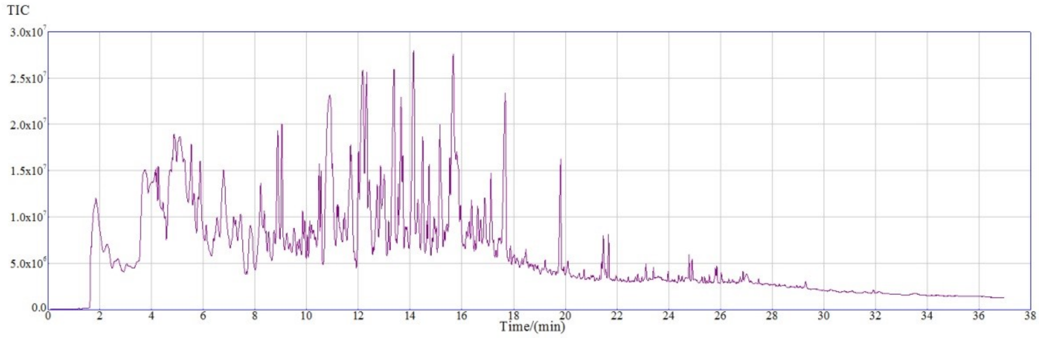

Figure S9. Py-GC-MS total ion chromatograms of PPs -Mo samples at 700 °C.

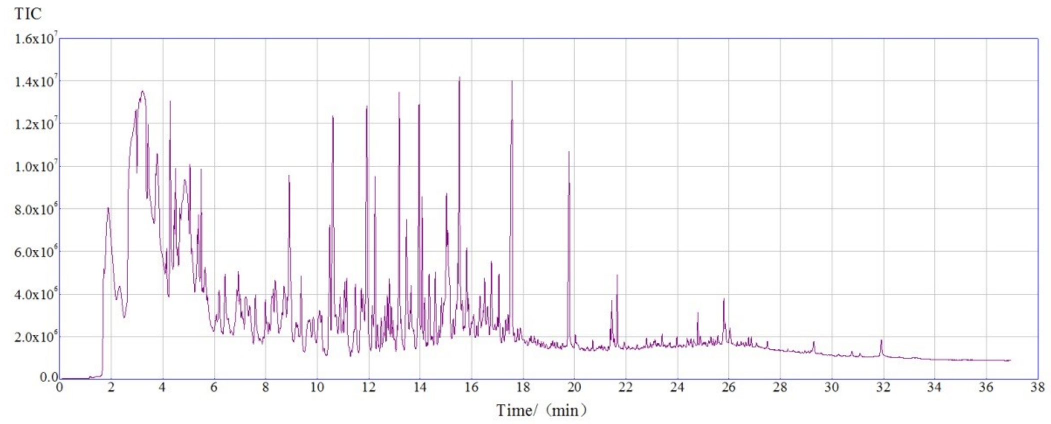

Figure S10 Py-GC-MS total ion chromatograms of PPs -Co3O4 samples at 700 ℃.

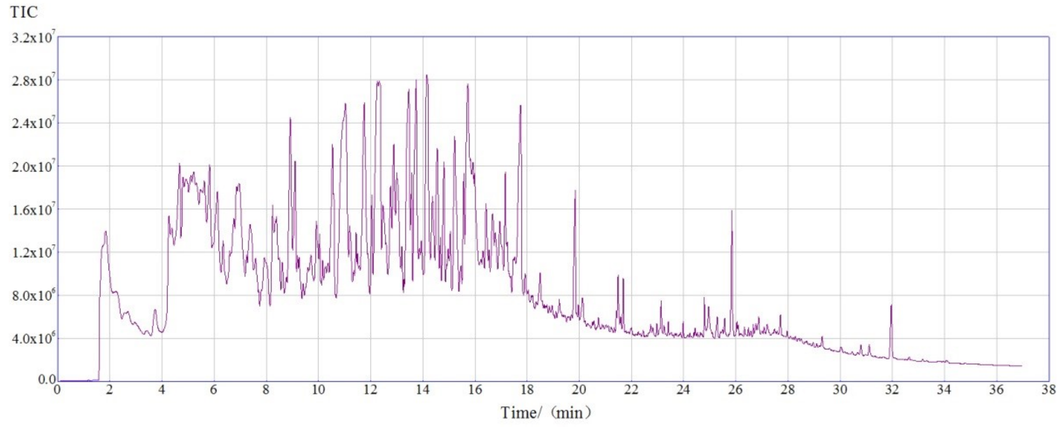

Figure S11 Py-GC-MS total ion chromatograms of PPs -Mo/Co3O4 (1:1) samples at 700 ℃.

Supplementary Tables

Table S1 The distribution area and production of peaches (Unit: ton)

Region	Area	Value	Region	Area	Value
**Asia**	Afghanistan	47935	**Europe**	Albania	19472
	Armenia	52247		Austria	2671
	Azerbaijan	26311		Bosnia and Herzegovina	9429
	Bhutan	960		Bulgaria	28879
	China	15217797		Croatia	3545
	Cyprus	2061		Czechia	962
	Democratic People's Republic of Korea	120985		France	184064
	Georgia	27500		Germany	562
	India	278417		Greece	968720
	Iran (Islamic Republic of)	645499		Hungary	23354
	Iraq	516		Italy	1090678
	Israel	50014		Malta	408
	Japan	113200		Montenegro	1241
	Jordan	43077		North Macedonia	13128
	Kazakhstan	2447		Poland	10583
	Kyrgyzstan	11990		Portugal	46666
	Lebanon	30749		Republic of Moldova	24660
	Nepal	11873		Romania	22633
	Pakistan	73843		Russian Federation	36500
	Palestine	2493		Serbia	73657
	Republic of Korea	205742		Slovakia	1564
	Syrian Arab Republic	44878		Slovenia	4457
	Tajikistan	29882		Spain	903809
	Turkey	789457		Switzerland	226
	Turkmenistan	29400		Ukraine	26380
	Uzbekistan	161930	**Oceania**	Australia	82407
	Yemen	17132		New Zealand	4008
**Africa**	Algeria	190420	**Americas**	Argentina	226000
	Cameroon	1064		Bolivia (Plurinational State of)	59642
	Egypt	246742		Brazil	219598
	Ethiopia	164		Canada	26931
	Kenya	232		Chile	319047
	Libya	14620		Colombia	29912
	Madagascar	11979		Ecuador	4206
	Malawi	12947		Guatemala	46005
	Morocco	157893		Mexico	160663

	Reunion	551		Paraguay	1299
	South Africa	152444		Peru	45473
	Tunisia	118662		United States of America	700350
				Uruguay	16414
	Zimbabwe	919		Venezuela (Bolivarian Republic of)	66250
**Total production (ton)**			24453425		

Table S2 UPLC/Q-TOF MS compositions of benzene extract from PPs samples.

No.	Retention time (min)	Content (%)	Compounds name
1	0.4	0.65	3,3',5,5'-tetramethoxy-anti
2	0.48	0.24	Norepinephrine
3	0.61	0.43	Ma Huang Kao Ning
4	2.52	1.85	Citron lactone
5	2.52	0.21	Danshen Xinyi B
6	2.85	0.31	3-methoxy-4-hydroxybenzoic acid
7	3.74	0.23	M-methoxycinnamic acid
8	3.9	0.70	Rhynchophylline
9	3.97	0.27	Pulmonene
10	4.08	0.21	3-isoaphine
11	4.09	0.18	Isobutyryl shikonin
12	4.17	0.43	Berberine
13	4.61	0.19	Glycyrrhizin
14	4.69	0.15	1-[(2E,4E)-2,4-dodecanoyl]pyrrole
15	4.85	0.20	Acanthopanax
16	5.37	1.73	Red coud
17	5.69	3.25	Pinoresinol dimethyl ether
18	6.59	0.24	Ginseng triol
19	6.71	0.47	Octadecedenoic acid
20	6.98	0.26	Ciryneone F
21	7.11	1.30	Curcumol
22	7.12	0.29	Methyl artemisinate
23	7.24	0.34	Dendrobium
24	7.3	0.24	Ginger (6-gingerol)
25	7.65	2.97	Cimifugin
26	7.65	0.24	7,7′ -dihydroxy-6,6′-dimethoxy-3,3′-dicoumarin
27	7.66	0.61	Schisandrin A
28	7.8	0.31	Alpha-monolinone
29	7.93	1.16	1-methyl-2-[(Z)-8-tetradecenyl]-4(1H)quinolone
30	7.93	0.32	Tryptophan
31	7.93	0.20	Bitter wood hemiacetal C
32	7.99	1.50	Schisandrin B (γ-Schisandrin)
33	8.57	1.89	Fruit chrysanthemum C
34	8.77	0.30	Rhynchophylline A
35	8.79	1.57	23-acetyl diarrhea lactone
36	8.79	1.24	Xing'an Cimicin E
37	8.79	0.21	Scutellaria
38	8.95	0.25	Streptavidin
39	8.96	4.31	Cis,cis-9,12-octadecadienol
40	8.96	1.74	(3,3-dimethyldecane)-benzene

41	8.96	0.29	Hidden goods
42	8.96	0.23	White and Lianfei C
43	8.98	0.51	Androstenediol
44	9.06	0.22	Psoralen dihydroflavone methyl ether
45	9.11	25.81	Monopalmitin
46	9.11	1.62	Lobetyol
47	9.11	1.00	Methyl 12-methyl-tetradecanoate
48	9.11	0.30	Gardening saponin F
49	9.11	0.23	Bitter Lactone L
50	9.12	0.44	Myristyl A
51	9.14	0.35	Deacetylated rice
52	9.19	1.55	Ferula fukanensis
53	9.2	0.32	Isohydrohydrogenine
54	9.26	0.34	Split larch lignan
55	9.34	0.22	Methyl 9,12-octadecadienoate
56	9.47	0.58	Benzyl phenylacetate
57	9.47	0.22	Phenylpropyl cinnamate
58	9.5	0.33	Palmitic monoglyceride
59	9.69	0.24	Alpha-hexadecyl glyceryl ether
60	9.71	1.01	Hydrogenated ginkgoic acid
61	9.84	9.01	Monostearin
62	9.84	0.50	Cis-9-octadecenal
63	10.07	0.40	epi-Kansenone
64	10.07	0.25	Rel-3R-methoxy-4S-furanogermacra-1E, 10(15)-dien-6-one
65	10.14	1.26	Methyl lucidum acid E
66	10.14	0.77	New matrine
67	10.14	0.51	Cniforin B
68	10.47	1.39	Three white grass lactam
69	10.51	1.55	Ergot-7,22-dien-3-ol
70	10.52	0.61	(E)-Hexadecyl-ferulate
71	10.56	0.37	Mutong acid
72	10.58	0.95	Methyl 12-hydroxyhydroabietic acid
73	10.6	1.35	Cardamom-4-ene-3,6-dione
74	10.71	1.19	4,22-dien-3-one decane
75	10.81	1.15	11α-hydroxytolic acid methyl ester
76	10.81	0.49	Paeonenolide H
77	10.84	9.25	3-hydroxystigmast-5-en-7-one

Table S3 UPLC/Q-TOF MS compositions of acetone extract from PPs samples.

No.	Retention time (min)	Content (%)	Compounds name
1	0.44	0.60	Isophora
2	0.44	0.25	Cnidimonal
3	0.44	0.22	Anthraquinone A
4	0.44	0.10	3,4-dihydroxyphenylethanol-3-O-β-D-glucopyranoside
5	0.44	0.09	Apigenin-7-O-acetyl-β-D-glucoside
6	0.45	1.08	6-aldehyde isoflavone A
7	0.48	0.15	Norepinephrine
8	0.48	0.12	Adenosine
9	0.49	0.21	Vitamin B5
10	0.61	0.26	Ma Huang Kao Ning
11	2.17	0.36	5-(butene-3-yne-1)-2,2'-bithiophene
12	2.35	3.63	Genipin
13	2.35	0.39	3-methoxy-4-hydroxybenzoic acid
14	2.35	0.28	Anisic acid
15	2.35	0.18	Methyl palmitate
16	2.35	0.12	Paeonol
17	2.56	1.03	Original cyanidin B4_1
18	2.57	0.25	Original cyanidin A2
19	2.61	0.96	Arecatannin B1
20	2.65	0.10	5,7-dihydroxychromanone-7-β-D-glucopyranoside
21	2.67	4.71	Cianidanol
22	2.67	0.74	Glucosinalbin
23	2.78	0.37	Arecatannin A2
24	2.78	0.13	Isoeugenol
25	2.81	1.00	Paclitaxel
26	2.83	0.15	Chloric acid
27	2.84	0.10	2,4,5-trimethoxybenzaldehyde
28	2.93	0.20	Artemisia annua
29	2.93	0.12	Kadsurenin B
30	2.96	0.13	Pseudo-mountain road year
31	2.98	0.21	Chlorpyrifos
32	3.09	0.55	Amygdalin
33	3.09	0.54	Anthraquinone I
34	3.09	0.18	Cyanoside
35	3.21	0.27	Salviamiltamide
36	3.22	0.15	Purpurin B
37	3.25	0.22	(+)-5,5'-dimethoxy larch
38	3.26	0.08	(3R,4R)-3,4-Dihydroxy-3-(3',4'-dimethoxybenzyl)-7-methoxy-chroman
39	3.31	0.82	2'-hydroxy-4',6'-dimethoxydihydrochalcone

40	3.31	0.45	2-carboxymethyl-3-isopentenyl-2,3-epoxy-1,4-naphthoquinone
41	3.31	0.18	Sangensu C
42	3.31	0.16	Danshen Xinyi B
43	3.31	0.14	(S)-4-methoxyxanthene
44	3.31	0.11	2,3,4,7-tetramethoxyphenanthrene
45	3.32	4.63	Pulmonene
46	3.32	1.97	Salvian phenolphthalein II
47	3.38	1.40	1,8-dihydroxy-3,5-dimethoxyfluorenone
48	3.38	1.23	5,7,2'-trihydroxydihydroflavone-4'-O-β-D-glucopyranoside
49	3.38	0.27	5,7,8,4'-tetrahydroxyflavone
50	3.38	0.11	Pseudohyperin
51	3.38	0.11	3,4-dihydroxy-6,7,3',4'-tetramethoxyflavone
52	3.4	0.15	Snake bed
53	3.41	0.31	3,3',5,5'-tetramethoxy-anti-
54	3.41	0.19	Obovatol
55	3.46	0.15	Acacia B
56	3.58	0.15	Myristyl B
57	3.6	0.42	Chinyl lactone alkenal
58	3.61	0.23	Alfalfa D
59	3.66	2.55	Red puromycin
60	3.66	2.53	5,7,4'-trihydroxy-8-C-β-D-glucose dihydroflavone carbon
61	3.66	0.15	5,7-dihydroxy-3'-methoxyflavone-4'-O-β-D-glucoside
62	3.73	0.12	Mustard aldehyde
63	3.75	0.24	M-methoxycinnamic acid
64	3.75	0.09	Flavonoid
65	3.91	0.28	Bitterwood S
66	3.91	0.11	Red melamine
67	3.98	0.24	Hydroxytanshinone IIA
68	3.98	0.21	Psorachalcone B (Corylifol B)
69	3.98	0.13	New psoralen isoflavone
70	4.04	0.10	Emodin
71	4.04	0.10	Isoflavone
72	4.04	0.08	Isomucronustyrene
73	4.09	0.79	Isobutyryl shikonin
74	4.09	0.13	Sophora flavescens
75	4.15	0.13	Decursidate
76	4.27	0.91	Josson
77	4.3	0.48	Cissogenin
78	4.3	0.31	1β-Hydroxycolartin
79	4.31	0.32	1-[(2E,4E)-2,4-decadienoyl]pyrrolidine
80	4.39	0.52	Three leucovorin
81	4.39	0.23	Methyl salicylate
82	4.39	0.22	Bitter mecoline F

83	4.42	0.46	Gardenin A
84	4.43	0.17	Sophora flavescens V
85	4.51	0.12	Apigenin-7-O-β-D-glucuronide
86	4.61	0.21	Alkaloid
87	4.61	0.15	Bakuchiol
88	4.61	0.10	9,16-dihydroxy-10,12,14-triene-octadecylate
89	4.62	0.88	Glycyrrhizin
90	4.62	0.18	Trans-acetic acid acetate
91	4.62	0.12	4,9-dimethoxy-1-vinyl-β-carboline
92	4.85	0.14	Acanthopanax
93	4.94	0.14	Isobutyric acid thyme ester
94	5.37	0.98	Red coud
95	5.69	1.47	Pinoresinol dimethyl ether
96	6.36	0.46	7β-(3-Ethyl-cis-crotonoyloxy)-14-hydroxynotonipetranone
97	6.71	1.01	Octadecedenoic acid
98	6.71	0.11	(3R)-Duartin
99	6.83	0.10	Levulin
100	7.11	0.61	Curcumol
101	7.15	0.18	Diaphora acid
102	7.65	1.48	Diisobutyl phthalate
103	7.65	0.16	Cimicin
104	7.8	0.15	Alpha-monolinone
105	7.93	0.89	Hydrogenated ginkgoic acid
106	7.93	0.70	Bitter wood hemiacetal C
107	7.93	0.48	Tryptophan
108	7.93	0.13	5,7,4'-trihydroxy-8,3'-diisopentenyl flavonoid
109	8.05	0.55	1-methyl-2-[(Z)-8-tetradecenyl]-4(1H)quinolone
110	8.37	0.10	5-dehydroindole
111	8.57	2.05	Fruit chrysanthemum C
112	8.77	0.16	Rhynchophylline A
113	8.79	0.11	Methyl lucidum acid E
114	8.8	0.18	23-acetyl diarrhea lactone
115	8.8	0.14	Xing'an Cimicin E
116	8.95	3.32	Cis,cis-9,12-octadecadienol
117	8.95	1.35	(3,3-dimethyldecane)-benzene
118	8.95	0.22	Streptavidin
119	8.95	0.18	Hidden goods
120	8.95	0.14	White and Lianfei C
121	8.96	0.12	Ningbeixin
122	8.96	0.08	Ganoderma alcohol A
123	8.98	0.26	Androstenediol
124	9.06	0.16	Psoralen dihydroflavone methyl ether
125	9.09	0.10	Deacetylated rice
126	9.1	13.20	Monopalmitin

127	9.1	0.54	Hexadecanoic acid
128	9.11	0.98	Lobetyol
129	9.11	0.13	Bitter Lactone L
130	9.12	0.24	Myristyl A
131	9.2	0.18	Isohydrohydrogenine
132	9.26	0.34	Split larch lignan
133	9.3	0.18	1-methyl-2-pentadecyl 4(1H) quinolone
134	9.41	1.02	4,22-dien-3-one decane
135	9.47	1.21	5-hydroxy-1,7-bisphenyl-3-heptanone
136	9.47	0.30	Benzyl phenylacetate
137	9.47	0.11	Phenylpropyl cinnamate
138	9.5	0.19	Palmitic monoglyceride
139	9.55	0.36	Diphenylamine
140	9.55	0.16	Yuanhufei base
141	9.6	0.11	Sitosterol-β-D-glucopyranoside-tetraacetate
142	9.69	0.14	Alpha-hexadecyl glyceryl ether
143	9.7	0.19	Oleanolic acid -28-O-β-D-glucopyranoside
144	9.8	0.81	Beer sterol
145	9.84	0.33	Cis-9-octadecenal
146	9.84	0.19	Ethyl palmitate
147	9.92	0.18	Saponins B
148	9.95	0.26	(E)-Hexadecyl-ferulate
149	9.99	0.19	Hexanol
150	10.07	1.42	Di(2-diethylhexyl) phthalate
151	10.07	0.32	epi-Kansenone
152	10.18	0.16	Cardamom-4-ene-3,6-dione
153	10.5	1.08	Ergot-7,22-dien-3-ol
154	10.53	0.40	Methyl 12-hydroxyhydroabietic acid
155	10.56	0.20	Mutong acid
156	10.6	0.22	Dandelion sterol acetate
157	10.62	1.08	(20S,24S)-3β-O-acetyl-20,25-epoxydammarane-24-ol
158	10.64	0.57	Wool sterol
159	10.7	0.15	Anti-ferulic acid n-octadecyl ester
160	10.8	0.71	11α-hydroxytolic acid methyl ester
161	10.8	0.28	Paeonenolide H
162	10.83	5.87	3-hydroxystigmast-5-en-7-one
163	10.94	0.57	Triolein
164	10.98	0.28	Dimethylquinoline

Table S4 UPLC/Q-TOF MS compositions of ethanol extract from PPs samples.

No.	Retention time (min)	Content (%)	Compounds name
1	0.43	0.22	Apigenin-7-O-β-D-glucuronide
2	0.44	0.36	Isophora
3	0.44	0.36	Dihydroxyisosorbate
4	0.44	0.21	Anthraquinone A
5	0.44	0.16	Cnidimonal
6	0.44	0.16	sucrose
7	0.44	0.13	3-O-4"-hydroxy-3", 5"-dimethoxybenzoyl chlorogenic acid methyl ester
8	0.44	0.12	3,4-dihydroxyphenylethanol-3-O-β-D-glucopyranoside
9	0.44	0.11	Hexahexanol
10	0.45	0.09	Wang does not leave flavonoid glycosides
11	0.46	0.25	6-aldehyde isoflavone A
12	0.48	0.18	Lotus root glycosides
13	0.48	0.10	Norepinephrine
14	0.60	0.26	Ma Huang Kao Ning
15	1.18	0.32	Adenosine
16	2.35	0.12	Genipin
17	2.56	1.28	Original cyanidin B4_1
18	2.62	0.93	Arecatannin B1
19	2.65	0.18	5,7-dihydroxychromanone-7-β-D-glucopyranoside
20	2.65	0.12	5,7-dihydroxychromanone
21	2.67	4.69	(+)-Catechin hydrate
22	2.67	0.65	Glucosinalbin
23	2.67	0.08	O-coumaric acid
24	2.68	0.44	Arecatannin A2
25	2.73	0.07	Carbafura-3-mannin
26	2.77	0.19	3-methoxy-4-hydroxybenzoic acid
27	2.78	0.12	Isoeugenol
28	2.83	0.16	Chloric acid
29	2.83	0.06	Methyl 3,4,5-trimethoxycinnamate
30	2.84	0.09	2,4,5-trimethoxybenzaldehyde
31	2.93	0.17	Artemisia annua
32	2.93	0.09	Kadsurenin B
33	2.93	0.06	Corchoionoside C
34	2.96	0.12	Pseudo-mountain road year
35	3.00	0.13	Snake bed
36	3.09	0.42	Anthraquinone I
37	3.09	0.19	Salviamiltamide
38	3.09	0.16	Cyanoside
39	3.22	0.52	Purpurin B

40	3.25	0.21	(+)-5,5'-dimethoxy larch
41	3.25	0.07	(3R,4R)-3,4-Dihydroxy-3-(3',4'-dimethoxybenzyl)-7-methoxy-chroman
42	3.26	0.09	6'-O-galloyl arbutin
43	3.29	0.17	Emodin
44	3.31	3.99	Pulmonene
45	3.31	1.61	Salvian phenolphthalein II
46	3.31	0.68	2'-hydroxy-4',6'-dimethoxydihydrochalcone
47	3.31	0.36	2-carboxymethyl-3-isopentenyl-2,3-epoxy-1,4-naphthoquinone
48	3.31	0.14	Danshen Xinyi B
49	3.31	0.12	(S)-4-methoxyxanthene
50	3.31	0.09	Sangensu C
51	3.36	0.13	Mururin A
52	3.38	1.57	1,8-dihydroxy-3,5-dimethoxyfluorenone
53	3.38	1.47	5,7,2'-trihydroxydihydroflavone-4'-O-β-D-glucopyranoside
54	3.38	0.26	5,7,8,4'-tetrahydroxyflavone
55	3.38	0.13	3,4-dihydroxy-6,7,3',4'-tetramethoxyflavone
56	3.38	0.07	Pseudohyperin
57	3.41	0.29	3,3',5,5'-tetramethoxy-anti-
58	3.41	0.17	Obovatol
59	3.42	0.26	Baoji Ig
60	3.44	0.09	3,7-Dihydroxy-4-methoxy-3-(3',4'-dimethoxybenzyl)-chroman
61	3.46	0.22	Acacia B
62	3.56	0.09	Isorhamnetin-3-glucoside
63	3.57	0.13	Bitter xylate H
64	3.57	0.07	Acetyl cinnamene
65	3.58	0.13	Myristyl B
66	3.58	0.08	4-hydroxy-3-butylphenyl peptide
67	3.60	0.38	Chinyl lactone alkenal
68	3.60	0.20	Alfalfa D
69	3.65	0.42	Original cyanidin A2
70	3.66	2.67	5,7,4'-trihydroxy-8-C-β-D-glucose dihydroflavone carbon
71	3.66	2.59	Red puromycin
72	3.66	0.16	5,7-dihydroxy-3'-methoxyflavone-4'-O-β-D-glucoside
73	3.73	0.14	(+)-1-hydroxy rosin phenol
74	3.73	0.08	Mustard aldehyde
75	3.74	0.20	Myristyl A
76	3.75	0.20	M-methoxycinnamic acid
77	3.91	0.38	Bitterwood S
78	3.91	0.11	Red melamine
79	3.98	0.46	Hydroxytanshinone IIA
80	3.98	0.43	Psorachalcone B (Corylifol B)

81	3.98	0.28	New psoralen isoflavone
82	4.04	0.08	Isoflavone
83	4.04	0.06	Isomucronustyrene
84	4.09	0.65	Isobutyryl shikonin
85	4.09	0.18	Sophora flavescens
86	4.09	0.11	Octanomycin
87	4.14	0.08	4-hydroxy-3-butylphenyl peptide
88	4.15	0.25	Decursidate
89	4.15	0.13	6-hydroxy-2-[2-(3'-methoxy-4'-hydroxyphenyl)ethyl]chromone
90	4.19	0.09	Isocalcin C
91	4.27	1.00	Josson
92	4.30	0.37	Cissogenin
93	4.30	0.23	1β-Hydroxycolartin
94	4.30	0.22	7β-(3-Ethyl-cis-crotonoyloxy)-14-hydroxynotonipetranone
95	4.34	0.06	Lonchocarpenin
96	4.39	0.56	Three leucovorin
97	4.39	0.28	Bitter mecoline F
98	4.39	0.23	Methyl salicylate
99	4.39	0.09	Psoralen isoflavone
100	4.39	0.06	Silver camphor B
101	4.42	0.49	Josson
102	4.42	0.44	Gardenin A
103	4.43	0.20	Sophora flavescens V
104	4.47	0.12	Alizarin
105	4.48	0.14	Methyl salicylate
106	4.61	0.11	Bakuchiol
107	4.61	0.09	Alkaloid
108	4.61	0.08	9,16-dihydroxy-10,12,14-triene-octadecylate
109	4.62	0.47	Glycyrrhizin
110	4.62	0.21	Carotenoid
111	4.62	0.12	Trans-acetic acid acetate
112	4.62	0.11	3-isoaphine
113	4.62	0.10	1-Acetyl-3-(methoxy-carbonyl)-β-carboline
114	5.37	0.64	Red coud
115	5.69	1.00	Pinoresinol dimethyl ether
116	6.71	0.56	Octadecedenoic acid
117	6.71	0.07	(3R)-Duartin
118	6.83	0.07	Levulin
119	7.65	0.27	Cimifugin
120	7.80	0.10	Alpha-monolinone
121	7.93	0.87	1-methyl-2-[(Z)-8-tetradecenyl]-4(1H)quinolone
122	7.93	0.55	Hydrogenated ginkgoic acid
123	7.93	0.37	Bitter wood hemiacetal C

124	7.93	0.28	Tryptophan
125	8.21	0.92	Arbutin
126	8.21	0.79	Amygdalin
127	8.21	0.32	Aconitine (new aconitine)
128	8.21	0.25	2,4,5-trimethoxybenzoic acid
129	8.21	0.21	Eucommia diol
130	8.21	0.11	Xin Kein Ib
131	8.21	0.10	Salvianolic acid A
132	8.56	1.18	Fruit chrysanthemum C
133	8.77	0.15	Rhynchophylline A
134	8.79	0.12	23-acetyl diarrhea lactone
135	8.79	0.11	Xing'an Cimicin E
136	8.79	0.08	Methyl lucidum acid E
137	8.95	1.47	Cis,cis-9,12-octadecadienol
138	8.95	0.65	(3,3-dimethyldecane)-benzene
139	8.95	0.11	Hidden goods
140	8.95	0.08	Streptavidin
141	8.96	0.13	Ningbeixin
142	9.06	0.07	Psoralen dihydroflavone methyl ether
143	9.10	10.98	Monopalmitin
144	9.10	0.42	Hexadecanoic acid
145	9.10	0.11	Gardening saponin F
146	9.11	0.63	Lobetyol
147	9.12	0.16	Hyodeoxycholic acid
148	9.14	0.13	Deacetylated rice
149	9.20	0.31	Ferula fukanensis
150	9.20	0.14	Isohydrohydrogenine
151	9.26	0.25	Split larch lignan
152	9.46	0.12	Phenylpropyl cinnamate
153	9.46	0.07	2-(2-phenylethyl) chromone
154	9.47	0.37	Benzyl phenylacetate
155	9.50	0.08	Palmitic monoglyceride
156	9.58	1.94	Phytolacca cerebroside
157	9.83	3.63	Monostearin
158	9.84	0.22	Cis-9-octadecenal
159	9.88	0.30	Beer sterol
160	9.95	0.19	(E)-Hexadecyl-ferulate
161	9.99	0.11	Hexanol
162	10.07	0.25	epi-Kansenone
163	10.50	2.70	Ergot-7,22-dien-3-ol
164	10.56	0.12	Mutong acid
165	10.59	0.24	Schisandra chinensis D
166	10.60	0.20	Cardamom-4-ene-3,6-dione
167	10.65	18.22	3-hydroxystigmast-5-en-7-one

168	10.71	2.55	4, 22-dien-3-one decane
169	10.72	0.50	Three white grass lactam
170	10.80	0.40	11α-hydroxytolic acid methyl ester
171	10.80	0.17	Paeonenolide H

Table S5 Classification of the UPLC/Q-TOF MS functional components in benzene extract

Component name	Content/ %	Function					
		Bioenergy	Biomedicine	Spices	Chemical raw materials	Food additive	Cosmetics
3-methoxy-4-hydroxybenzoic acid	0.31	/	/	Yes	Yes	/	/
M-methoxycinnamic acid	0.23	/	/	Yes	/	/	Yes
Rhynchophylline	0.70	/	Yes	/	/	/	/
Red coud	1.73	/	Yes	/	/	/	/
Ginseng triol	0.24	/	Yes	/	/	/	/
Octadecedenoic acid	0.47	Yes	/	/	/	/	/
Ginger (6-gingerol)	0.24	/	Yes	/	/	/	/
Cimicin	2.97	/	Yes	/	/	/	/
Schisandrin A	0.61	/	Yes	/	/	/	/
1-methyl-2-[(Z)-8-tetradecenyl]-4(1H)quinolone	1.16	Yes	/	/	/	/	/
Tryptophan	0.32	/	Yes	/	/	/	/
Androstenediol	0.51	/	Yes	/	/	/	/
Monopalmitate	25.81	/	Yes	/	/	Yes	/
Benzyl phenylacetate	0.58	/	/	Yes	/	Yes	/
Glyceryl monostearate	9.01	/	Yes	Yes	Yes	Yes	Yes

Table S6 Classification of the UPLC/Q-TOF MS functional components in acetone extract

Component name	Content/ %	Function					
		Bioenergy	Biomedicine	Spices	Chemical raw materials	Food additive	Cosmetics
Adenosine	0.12	/	Yes	/	/	/	/
Genipin	3.63	/	Yes	/	/	/	/
3-methoxy-4-hydroxybenzoic acid	0.39	/	/	Yes	Yes	/	/
Paeonol	0.12	/	Yes	/	/	/	/
Catechin	4.71	/	Yes	/	/	Yes	/
Isoeugenol	0.13	/	/	Yes	/	Yes	/
2,4,5-trimethoxybenzaldehyde	0.10	/	Yes	/	/	/	/
Amygdalin	0.55	/	Yes	/	/	/	/
M-methoxycinnamic acid	0.24	/	/	Yes	/	/	Yes
Flavonoid	0.09	/	Yes	/	Yes	/	/
New psoralen isoflavone	0.13	/	Yes	/	/	/	/
Red coud	0.98	/	Yes	/	/	/	/
Octadecedenoic acid	1.01	Yes	/	/	/	/	/
Diisobutyl phthalate	1.48	/	/	/	Yes	/	/
Cimicin	0.16	/	Yes	/	/	/	/
Tryptophan	0.48	/	Yes	/	/	/	/
Androstenediol	0.26	/	Yes	/	/	/	/
Monopalmitate	13.20	/	Yes	/	/	Yes	/
Benzyl phenylacetate	0.30	/	/	Yes	/	Yes	/
Diphenylamine	0.36	Yes	Yes	/	Yes	/	/
Ethyl palmitate	0.19	/	/	Yes	/	Yes	/
Triolein	0.57	/	Yes	/	Yes	/	/
Dimethylquinoline	0.28	/	/	/	/	/	/

Table S7 Classification of the UPLC/Q-TOF MS functional components in ethanol extract

Component name	Content /%	Function					
		Bioenergy	Biomedicine	Spices	Chemical raw materials	Food additive	Cosmetics
sucrose	0.16	/	Yes	Yes	/	Yes	Yes
O-coumaric acid	0.08	/	Yes	/	/	/	/
3-methoxy-4-hydroxybenzoic acid	0.19	/	/	Yes	Yes	/	/
M-methoxycinnamic acid	0.20	/	/	Yes	/	/	Yes
Red coud	0.64	/	Yes	/	/	/	/
Kommon dimethyl ether	1.00	/	/	/	/	/	/
Octadecedenoic acid	0.56	Yes	/	/	/	/	/
(3R)-Duartin	0.07	/	/	/	/	/	/
Levulin	0.07	/	/	/	/	/	/
Cimicin	0.27	/	Yes	/	/	/	/
Alpha-monolinone	0.10	/	/	/	/	/	/
Tryptophan	0.28	/	Yes	/	/	/	/
Aconitine (new aconitine)	0.32	/	Yes	/	/	/	/
2,4,5-trimethoxybenzoic acid	0.25	/	Yes	/	/	/	/
Monopalmitate	10.98	/	Yes	/	/	Yes	/
Benzyl phenylacetate	0.37	/	/	Yes	/	Yes	/

Table S8 Py-GC-MS components of non-catalytic PPs samples at 550 °C.

No.	Retention time (min)	Relative content (%)	Compounds name
1	2.72	7.22	Acetic acid
2	4.77	0.96	Furfural
3	5.25	1.06	1,6:2,3-Dianhydro-4-O-acetyl-.beta.-d-gulopyranose
4	5.64	0.17	Bicyclo[4.2.0]octa-1,3,5-triene
5	6.89	0.53	2-Furancarboxaldehyde, 5-methyl-
6	7.27	0.35	Phenol
7	7.46	0.27	Phenol
8	8.28	0.44	Glycerin
9	8.48	1.21	Glycerin
10	8.65	0.63	Glycerin
11	8.92	1.36	Phenol, 2-methoxy-
12	9.14	0.36	2-Nonen-1-ol, (E)-
13	9.45	0.24	2-Cyclopenten-1-one, 3-ethyl-2-hydroxy-
14	9.60	0.28	4-Pyridinol
15	9.85	0.38	4H-Pyran-4-one, 2,3-dihydro-3,5-dihydroxy-6-methyl-
16	10.31	0.08	1H-Pyrrole, 1-methyl-
17	10.51	0.38	Creosol
18	11.54	0.22	1,2-Benzenediol, 3-methoxy-
19	11.74	0.13	Phenol, 4-ethyl-2-methoxy-
20	11.98	0.12	Hydroquinone
21	12.08	0.27	1,2-Benzenediol, 4-methyl-
22	12.27	0.25	2-Methoxy-4-vinylphenol
23	12.74	0.42	Phenol, 2,6-dimethoxy-
24	12.90	0.30	Pyridine, 2-(1-methyl-2-pyrrolidinyl)-
25	13.62	0.07	2,3-Dimethylhydroquinone
26	13.99	0.18	3,5-Dimethoxy-4-hydroxytoluene
27	14.09	0.29	Phenol, 2-methoxy-4-(1-propenyl)-, (Z)-
28	14.49	0.09	2'-Amino-4'-methoxyacetanilide
29	14.58	0.17	5-Hepten-3-yn-2-ol, 6-methyl-5-(1-methylethyl)-
30	14.96	1.30	phosphorane, (3,5-dimethylphenyl)dimethyl-, oxide
31	15.08	0.30	2-Propanone, 1-(4-hydroxy-3-methoxyphenyl)-
32	15.48	0.72	2,3,5,6-Tetrafluoroanisole
33	15.79	0.25	4-Amino-2-nitro-benzaldehyde oxime
34	15.89	0.27	Phenol, 2,6-dimethoxy-4-(2-propenyl)-
35	15.97	0.37	Thioguanine
36	16.48	1.15	(E)-2,6-Dimethoxy-4-(prop-1-en-1-yl)phenol
37	16.79	1.01	Benzaldehyde, 4-butoxy-
38	17.09	1.44	(E)-2,6-Dimethoxy-4-(prop-1-en-1-yl)phenol
39	17.45	0.58	8-Hydroxyquinoline
40	17.59	0.81	4-((1E)-3-Hydroxy-1-propenyl)-2-methoxyphenol

41	17.75	0.72	Tetradecanoic acid
42	17.80	0.63	Phenol, 2,6-dimethyl-4-nitro-
43	18.60	0.91	Cyclohexane, 3,4-bis(1-methylethenyl)-1,1-dimethyl-
44	18.80	0.89	Pentadecanoic acid
45	19.13	1.24	1,1'-Biphenyl, 4-methyl-
46	19.24	0.42	4-Nitro-5,6,7,8-tetrahydronaphthalen-1-ol
47	19.34	0.49	1H,3H-Pyrano[3,4-c]pyran-5-carboxaldehyde, 4,4a,5,6-tetrahydro-6-methyl-1-oxo-, [4as-(4a.alpha.,5.alpha.,6.beta.)]-
48	19.44	0.37	Hexadecenenitrile
49	19.63	1.34	Cyclopropaneoctanal, 2-octyl-
50	19.85	2.92	n-Hexadecanoic acid
51	20.09	0.43	2(1H)-Benzocyclooctenone, decahydro-4a-methyl-, trans-(-)-
52	20.18	0.82	2,5-Dimethoxybenzoic acid
53	20.33	0.97	9H-Pyrido[3,4-b]indole
54	20.47	0.33	Phosphoric acid, dimethyl methylphenyl ester
55	20.59	0.82	Cyclopentadecanone, 2-hydroxy-
56	20.67	0.56	1,4-Methanobenzocyclodecene, 1,2,3,4,4a,5,8,9,12,12a-decahydro-
57	20.79	0.66	9-Octadecynoic acid
58	20.91	0.49	2-Cyclohexen-1-one, 3-(3-hydroxybutyl)-2,4,4-trimethyl-
59	20.99	0.79	cis-9-Hexadecenal
60	21.55	4.86	9,12-Octadecadienoic acid (Z,Z)-
61	21.73	0.82	Cyclopentadecanone, 2-hydroxy-
62	21.84	0.72	Cyclohexene, 1-pentyl-4-(4-propylcyclohexyl)-
63	21.92	2.15	Tetradecanamide
64	22.16	0.99	9,12-Octadecadienoic acid (Z,Z)-
65	22.29	2.53	9,12-Octadecadienoic acid (Z,Z)-
66	22.61	0.40	Cyclohexene, 1-pentyl-4-(4-propylcyclohexyl)-
67	22.67	0.61	Oxacyclohexadecan-2-one
68	22.86	1.60	3-Tridecylphenol
69	23.00	0.59	Nitroscanate
70	23.11	0.81	Z-11-Pentadecenol
71	23.28	1.37	7-Isopropyl-2,10-dimethyl-1,5-dithia-spiro[5.5]undecane 1-oxide
72	23.43	1.75	[1,2,4]Triazolo[3,4-b][1,3,4]thiadiazole, 3-methyl-6-(4-propoxyphenyl)-
73	23.69	0.89	Oxacyclohexadecan-2-one
74	23.92	1.78	2(1H)-Naphthalenone, octahydro-4a-methyl-7-(1-methylethyl)-, (4a.alpha.,7.beta.,8a.beta.)-
75	24.16	0.30	Heptadecanolide
76	24.23	0.58	7,10-Hexadecadienoic acid, methyl ester
77	24.44	2.49	(Z)-3-(pentadec-8-en-1-yl)phenol
78	24.52	1.26	(Z)-3-(pentadec-8-en-1-yl)phenol
79	24.68	1.12	Hexadecanoic acid, 2-hydroxy-1-(hydroxymethyl)ethyl ester
80	24.82	0.81	9-t-Butyltricyclo[4.2.1.1(2,5)]decane-9,10-diol
81	25.01	0.67	Ledene oxide-(I)
82	25.08	0.80	Z-11-Pentadecenol

83	25.23	0.29	Heptadecanolide
84	25.32	0.71	6-Octadecenoic acid
85	25.43	1.52	Cyclopropaneoctanal, 2-octyl-
86	25.86	1.05	Heptadecanolide
87	26.06	2.42	1-Tricosene
88	26.13	1.57	(Z)-3-(Heptadec-10-en-1-yl)phenol
89	26.41	0.89	Cyclopentadecanone, 2-hydroxy-
90	26.54	0.67	2,5-Furandione, 3-dodecyl-
91	26.78	1.27	Eicosane
92	26.90	2.40	Squalene
93	27.38	0.54	Fumaric acid, pent-4-en-2-yl tridecyl ester
94	27.52	3.93	Heptadecane
95	27.92	1.01	.delta.-Tocopherol
96	28.31	1.04	Eicosane
97	29.24	1.12	Heptadecane
98	29.35	1.05	Stigmasta-3,5-diene
99	29.61	1.45	dl-.alpha.-Tocopherol
100	30.02	0.77	1-Bromo-11-iodoundecane
101	30.52	0.15	1-Benzazirene-1-carboxylic acid, 2,2,5a-trimethyl-1a-[3-oxo-1-butenyl] perhydro-, methyl ester
102	30.60	0.24	1-Benzazirene-1-carboxylic acid, 2,2,5a-trimethyl-1a-[3-oxo-1-butenyl] perhydro-, methyl ester
103	31.16	0.67	4-Dehydroxy-N-(4,5-methylenedioxy-2-nitrobenzylidene)tyramine
104	31.42	0.20	1-Benzazirene-1-carboxylic acid, 2,2,5a-trimethyl-1a-[3-oxo-1-butenyl] perhydro-, methyl ester
105	31.59	0.37	Eicosane
106	31.71	0.28	1-Benzazirene-1-carboxylic acid, 2,2,5a-trimethyl-1a-[3-oxo-1-butenyl] perhydro-, methyl ester
107	32.00	0.71	.beta.-Sitosterol
108	32.34	0.22	1-Benzazirene-1-carboxylic acid, 2,2,5a-trimethyl-1a-[3-oxo-1-butenyl] perhydro-, methyl ester
109	32.58	0.19	1-Benzazirene-1-carboxylic acid, 2,2,5a-trimethyl-1a-[3-oxo-1-butenyl] perhydro-, methyl ester
110	32.70	0.14	1-Benzazirene-1-carboxylic acid, 2,2,5a-trimethyl-1a-[3-oxo-1-butenyl] perhydro-, methyl ester
111	32.81	0.17	1-Benzazirene-1-carboxylic acid, 2,2,5a-trimethyl-1a-[3-oxo-1-butenyl] perhydro-, methyl ester
112	33.80	0.23	1-Benzazirene-1-carboxylic acid, 2,2,5a-trimethyl-1a-[3-oxo-1-butenyl] perhydro-, methyl ester
113	34.04	0.39	1-Benzazirene-1-carboxylic acid, 2,2,5a-trimethyl-1a-[3-oxo-1-butenyl] perhydro-, methyl ester

Table S9 Py-GC-MS components of PPs-Mo samples at 550 °C.

No.	Retention time (min)	Content (%)	Compounds name
1	1.42	0.00	Acetic acid, hydroxy[(1-oxo-2-propenyl)amino]-
2	2.93	12.43	Acetic acid
3	4.78	3.87	Furfural
4	5.58	0.70	Cyclopent-4-ene-1,3-dione
5	5.99	0.22	3-Furaldehyde
6	6.09	0.50	3-Hexene, (Z)-
7	6.34	0.52	4,5-Dihydro-2-methylimidazole-4-one
8	6.46	0.50	Cyclohexanone
9	6.72	0.10	3-Furanmethanol
10	6.88	1.11	Benzaldehyde
11	7.26	0.45	Phenol
12	7.46	0.13	Phenol
13	7.55	0.87	Oxazolidine, 2,2-diethyl-3-methyl-
14	8.11	1.80	Glycerin
15	8.27	0.86	Glycerin
16	8.48	1.52	Glycerin
17	8.60	0.70	Glycerin
18	8.91	1.51	Phenol, 2-methoxy-
19	9.45	0.41	4-Pyridinol
20	9.55	0.30	4-Pyridinol
21	9.86	0.31	4H-Pyran-4-one, 2,3-dihydro-3,5-dihydroxy-6-methyl-
22	10.50	0.37	Creosol
23	11.12	0.23	1(2H)-Naphthalenone, octahydro-, cis-
24	11.54	0.11	meta-Methoxybenzenethiol
25	11.74	0.12	Phenol, 4-ethyl-2-methoxy-
26	11.98	0.13	Hydroquinone
27	12.07	0.27	1,2-Benzenediol, 4-methyl-
28	12.26	0.28	2-Methoxy-4-vinylphenol
29	12.74	0.29	Phenol, 2,6-dimethoxy-
30	12.83	0.32	Pyridine, 3-(1-methyl-2-pyrrolidinyl)-, (S)-
31	13.39	0.10	.beta.-D-Glucopyranose, 1,6-anhydro-
32	13.47	0.25	Vanillin
33	13.98	0.08	3,5-Dimethoxy-4-hydroxytoluene
34	14.09	0.27	Phenol, 2-methoxy-4-(1-propenyl)-
35	14.57	0.20	Ethanone, 1-[4-(methylthio)phenyl]-
36	14.83	0.42	.beta.-D-Glucopyranose, 1,6-anhydro-
37	14.95	0.22	phosphorane, (3,5-dimethylphenyl)dimethyl-, oxide
38	15.07	0.33	2-Propanone, 1-(4-hydroxy-3-methoxyphenyl)-
39	15.47	0.51	2,3,5,6-Tetrafluoroanisole
40	15.88	0.19	Phenol, 2,6-dimethoxy-4-(2-propenyl)-

41	15.96	0.14	4-Propyl-1,1'-diphenyl
42	16.52	1.22	Benzenepropanol, 4-hydroxy-3-methoxy-
43	16.62	0.40	Benzaldehyde, 4-hydroxy-3,5-dimethoxy-
44	16.78	0.84	Benzaldehyde, 4-butoxy-
45	17.08	1.41	(E)-2,6-Dimethoxy-4-(prop-1-en-1-yl)phenol
46	17.43	0.32	1,1'-Biphenyl, 4-(1-methylethyl)-
47	17.57	1.36	4-((1E)-3-Hydroxy-1-propenyl)-2-methoxyphenol
48	17.72	0.45	Tetradecanoic acid
49	18.04	0.85	Pinonic acid
50	18.78	0.73	Pentadecanoic acid
51	19.11	0.32	Diphenylmethane
52	19.22	0.33	Alloaromadendrene oxide-(2)
53	19.61	0.86	Hexadecenoic acid, Z-11-
54	19.83	2.71	n-Hexadecanoic acid
55	20.04	0.23	2(1H)-Benzocyclooctenone, decahydro-4a-methyl-, trans-(-)-
56	20.13	0.70	N-(5H-Indeno[1,2-b]pyridin-4-yl)-malonamic acid ethyl ester
57	20.30	0.75	9H-Pyrido[3,4-b]indole
58	20.42	0.33	[1,2,4]Triazolo[1,5-a]pyrimidin-5-ol, 7-methyl-6-nitro-
59	20.57	0.51	9-Hexadecenoic acid
60	20.89	0.31	Caparratriene
61	20.98	0.47	cis-9-Hexadecenal
62	21.06	0.22	9-Borabicyclo[3.3.1]nonane, 9-cyclooctyl-
63	21.12	0.27	2,6,10-Dodecatrien-1-ol, 3,7,11-trimethyl-, acetate, (E,E)-
64	21.21	0.45	Spiro[cyclopropane-1,8'(1H')[3a.6]methano[3ah]cyclopentacyclooctten]-10'-one, octahydro-, (3'as,6'R,9'ar)-
65	21.29	0.26	Tricyclo[4.2.1.1(2,5)]decane
66	21.51	3.00	9,17-Octadecadienal, (Z)-
67	21.71	0.98	Octadecanoic acid
68	21.81	0.43	2(1H)-Naphthalenone, octahydro-4a-methyl-7-(1-methylethyl)-, (4a.alpha.,7.beta.,8a.beta.)-
69	21.90	1.35	2(1H)-Naphthalenone, octahydro-4a-methyl-7-(1-methylethyl)-, (4a.alpha.,7.beta.,8a.beta.)-
70	22.15	0.73	Azuleno[6,5-b]furan-2,6(3H,4H)-dione, 3a,7,7a,8,9,9a-hexahydro-4-hydroxy-3,5,8-trimethyl-, [3R-(3.alpha.,3a.alpha.,4.alpha.,7a.beta.,8.beta.,9a.alpha.)]-
71	22.28	0.65	9,12-Octadecadienoic acid (Z,Z)-
72	22.35	0.47	Hexa-1,3,5-triene, 3-methyl-1,6-diphenyl-
73	22.46	0.67	Z-9-Pentadecenol
74	22.67	0.98	1,2-Longidione
75	22.76	0.37	Z-11-Pentadecenol
76	22.85	0.75	1,4,6-Trimethyl-1,2,3,3a,4,7,8,8a-octahydro-4,7-ethanoazulene
77	22.99	0.53	1,2,4-Triazolo[4,3-b]pyridazine, 6,8-diphenyl-
78	23.27	1.28	(+-)-2-Phenethanamine, 1-methyl-N-vanillyl-
79	23.42	1.68	7,10-Hexadecadienoic acid, methyl ester

80	23.68	0.87	Guanidine, N-[3-[(2-bromophenyl)amino]-1-propenyl]-
81	23.89	1.45	1,3-Dioxolo[4,5-c]pyran, tetrahydro-, 7-acetoxy-7-allyl-2,2-dimethyl-4-(3-methoxycarbonyl-2-methylallyl)-
82	24.09	0.26	Oxacycloheptadecan-2-one
83	24.14	0.26	Oxacycloheptadecan-2-one
84	24.22	0.67	7,10-Hexadecadienoic acid, methyl ester
85	24.43	1.73	(Z)-3-(pentadec-8-en-1-yl)phenol
86	24.51	1.08	(Z)-3-(pentadec-8-en-1-yl)phenol
87	24.68	1.27	Hexadecanoic acid, 2-hydroxy-1-(hydroxymethyl)ethyl ester
88	24.82	0.82	Z-11-Pentadecenol
89	25.08	1.58	2,5-Furandione, 3-dodecyl-
90	25.32	1.07	Batilol
91	25.72	0.34	Heptadecanolide
92	25.86	0.82	n-Propyl 9-octadecenoate
93	26.06	2.30	Fumaric acid, 2-chloropropyl dodecyl ester
94	26.13	1.94	(Z)-3-(Heptadec-10-en-1-yl)phenol
95	26.40	0.60	Cyclopentadecanone, 2-hydroxy-
96	26.58	0.59	1-Bromo-11-iodoundecane
97	26.73	0.93	Dichloro[(trichloromethyl)thio]methanesulfenyl chloride
98	26.79	0.52	Cyclopentadecanone, 2-hydroxy-
99	26.90	1.37	Squalene
100	26.99	1.41	Cyclohexene, 4-(4-ethylcyclohexyl)-1-pentyl-
101	27.38	0.46	Fumaric acid, pent-4-en-2-yl tridecyl ester
102	27.52	1.91	Heptadecane
103	27.92	1.15	.delta.-Tocopherol
104	28.14	0.37	1-Benzazirene-1-carboxylic acid, 2,2,5a-trimethyl-1a-[3-oxo-1-butenyl] perhydro-, methyl ester
105	28.31	1.26	Eicosane
106	28.83	0.73	.gamma.-Tocopherol
107	29.24	1.67	Octadecane
108	29.35	1.53	Stigmasta-3,5-diene
109	29.61	1.39	Vitamin E
110	31.09	0.22	2-Ethylacridine
111	31.59	0.42	Eicosane
112	31.73	0.35	2-Ethylacridine
113	32.00	1.12	.gamma.-Sitosterol
114	32.20	0.30	2-Ethylacridine
115	32.59	0.19	2-Ethylacridine
116	32.81	0.19	2-Ethylacridine
117	32.94	0.21	2-Ethylacridine
118	33.12	0.27	2-Ethylacridine
119	33.41	0.15	2-Ethylacridine
120	33.59	0.08	2-Ethylacridine

121	33.80	0.12	1,2-Bis(trimethylsilyl)benzene
122	34.04	0.07	1,4-Bis(trimethylsilyl)benzene
123	34.16	0.07	2-Ethylacridine
124	35.00	0.07	1,2-Bis(trimethylsilyl)benzene
125	35.15	0.03	2-Ethylacridine
126	35.24	0.02	2-Ethylacridine
127	35.42	0.00	2-Ethylacridine
128	35.60	0.01	1,2-Bis(trimethylsilyl)benzene

Table S10 Py-GC-MS components of PPs-Co_3O_4 samples at 550 °C.

No.	Retention time (min)	Content (%)	Compounds name
1	1.49	0.01	1-Pentanol, 4-amino-
2	3.02	7.46	Acetic acid
3	3.31	10.45	Acetic acid
4	4.82	7.47	Furfural
5	5.10	0.96	Furfural
6	5.54	2.00	2-Propanone, 1-(acetyloxy)-
7	6.10	0.88	Methanone, dicyclopropyl-
8	6.24	0.93	1H-Imidazole, 4,5-dihydro-2-methyl-
9	6.50	1.50	2-Cyclopenten-1-one, 2-hydroxy-
10	6.60	0.93	1,2-Cyclopentanedione
11	6.92	0.83	Benzaldehyde
12	7.05	0.71	2-Cyclopenten-1-one, 3-methyl-
13	7.32	0.71	Phenol
14	8.05	0.66	2-Cyclopenten-1-one, 2-hydroxy-3-methyl-
15	8.32	0.75	dl-Threitol
16	8.66	2.19	Glycerin
17	8.95	2.49	Phenol, 2-methoxy-
18	9.20	0.78	2-Formylhistamine
19	9.40	0.45	3-Hydroxypyridine monoacetate
20	9.77	0.50	3-Pyridinol
21	9.97	0.33	4H-Pyran-4-one, 2,3-dihydro-3,5-dihydroxy-6-methyl-
22	10.08	0.27	1,3-Dimethylimidazole-2(3H)-thione
23	10.52	0.84	Creosol
24	10.82	1.48	Catechol
25	11.20	0.80	5-Hydroxymethylfurfural
26	11.60	0.26	1,2-Benzenediol, 3-methoxy-
27	11.74	0.24	Phenol, 4-ethyl-2-methoxy-
28	11.80	0.12	Ethanone, 1-(2,5-dihydroxyphenyl)-
29	12.28	1.21	2-Methoxy-4-vinylphenol
30	12.76	0.56	Phenol, 2,6-dimethoxy-
31	12.83	0.38	Phenol, 2-methoxy-3-(2-propenyl)-
32	13.36	0.24	2-Cyclopenten-1-one, 2,3,4,5-tetramethyl-
33	13.51	1.71	Vanillin
34	14.00	0.23	3,5-Dimethoxy-4-hydroxytoluene
35	14.11	1.12	trans-Isoeugenol
36	14.22	0.45	Phenol, 2-methoxy-4-propyl-
37	14.52	0.23	Benzene, 1-methyl-4-[(methylthio)ethynyl]-
38	14.60	0.70	Apocynin
39	14.80	0.17	1,2-Dimethoxy-4-(methoxymethyl)benzene
40	14.96	0.46	phosphorane, (3,5-dimethylphenyl)dimethyl-, oxide

41	15.10	0.59	2-Propanone, 1-(4-hydroxy-3-methoxyphenyl)-
42	15.49	1.01	2,3,5,6-Tetrafluoroanisole
43	15.66	0.36	4-(1-Hydroxyallyl)-2-methoxyphenol
44	15.79	0.43	Butyrovanillone
45	15.89	0.29	Phenol, 2,6-dimethoxy-4-(2-propenyl)-
46	15.97	0.33	1-(2,5-Dimethoxyphenyl)-propanol
47	16.20	0.19	Benzoic acid, 3-hydrazino-4-methyl-
48	16.48	0.29	(E)-2,6-Dimethoxy-4-(prop-1-en-1-yl)phenol
49	16.55	0.77	Benzenepropanol, 4-hydroxy-3-methoxy-
50	16.65	0.48	Benzaldehyde, 4-hydroxy-3,5-dimethoxy-
51	16.83	1.09	4-((1E)-3-Hydroxy-1-propenyl)-2-methoxyphenol
52	16.95	0.24	2-Allyl-1,4-dimethoxy-3-methyl-benzene
53	17.10	1.20	(E)-2,6-Dimethoxy-4-(prop-1-en-1-yl)phenol
54	17.32	0.35	Imidazo[5,1-f][1,2,4]triazine-2,7-diamine, 4,5-dimethyl-
55	17.45	0.38	Ethanone, 1-(4-hydroxy-3,5-dimethoxyphenyl)-
56	17.62	3.26	4-((1E)-3-Hydroxy-1-propenyl)-2-methoxyphenol
57	17.73	0.72	4-((1E)-3-Hydroxy-1-propenyl)-2-methoxyphenol
58	17.82	1.32	Phenol, 2,6-dimethyl-4-nitro-
59	18.12	0.20	9-Ethoxy-10-oxatricyclo[7.2.1.0(1,6)]dodecan-11-one
60	18.19	0.20	9-Ethoxy-10-oxatricyclo[7.2.1.0(1,6)]dodecan-11-one
61	18.27	0.31	4-((1E)-3-Hydroxy-1-propenyl)-2-methoxyphenol
62	18.78	0.63	Pentadecanoic acid
63	18.96	0.21	7-Methyl-1,2-dihydroquinoxalin-2-one
64	19.06	0.10	Phenol, 4-(ethoxymethyl)-2-methoxy-
65	19.22	0.66	1-Dodecanethiol
66	19.50	0.12	4-Nitro-5,6,7,8-tetrahydronaphthalen-1-ol
67	19.61	0.72	Palmitoleic acid
68	19.84	1.72	n-Hexadecanoic acid
69	20.31	0.51	9H-Pyrido[3,4-b]indole
70	20.56	0.52	Cyclopentadecanone, 2-hydroxy-
71	20.77	0.30	Heptadecanoic acid
72	20.90	0.20	2(1H)-Naphthalenone, octahydro-4a-methyl-7-(1-methylethyl)-, (4a.alpha.,7.beta.,8a.beta.)-
73	20.98	0.15	Heptadecanenitrile
74	21.06	0.12	Spiro[4.5]decan-7-one, 1,8-dimethyl-8,9-epoxy-4-isopropyl-
75	21.13	0.21	Alloaromadendrene oxide-(2)
76	21.21	0.26	2(1H)-Benzocyclooctenone, decahydro-4a-methyl-, trans-(-)-
77	21.50	1.39	trans-13-Octadecenoic acid
78	21.70	0.49	Octadecanoic acid
79	21.81	0.18	Z-11-Pentadecenol
80	21.89	0.63	2(1H)-Naphthalenone, octahydro-4a-methyl-7-(1-methylethyl)-, (4a.alpha.,7.beta.,8a.beta.)-
81	22.14	0.26	1H-Benzocyclohepten-7-ol, 2,3,4,4a,5,6,7,8-octahydro-1,1,4a,7-tetramethyl-, cis-

82	22.28	0.48	9,12-Octadecadienoic acid (Z,Z)-
83	22.46	0.33	E-11-Hexadecenal
84	22.59	0.18	2-((2R,4aR,8aR)-4a,8-Dimethyl-1,2,3,4,4a,5,6,8a-octahydronaphthalen-2-yl)acrylaldehyde
85	22.65	0.15	1-Naphthalenepropanol, .alpha.-ethenyldecahydro-.alpha.,5,5,8a-tetramethyl-2-methylene-, [1S-[1.alpha.(S*),4a.beta.,8a.alpha.]]-
86	22.76	0.25	Z-11-Pentadecenol
87	22.85	0.35	Carbamic acid, methyl-, 3-methylphenyl ester
88	22.99	0.22	Spiro[4.5]decan-7-one, 1,8-dimethyl-8,9-epoxy-4-isopropyl-
89	23.10	0.45	Z-11-Pentadecenol
90	23.28	0.45	(E)-15,16-Dinorlabda-8(17),12-dien-14-al
91	23.42	0.67	2(1H)-Naphthalenone, octahydro-4a-methyl-7-(1-methylethyl)-, (4a.alpha.,7.beta.,8a.beta.)-
92	23.68	0.36	Cyclohexene, 4-(4-ethylcyclohexyl)-1-pentyl-
93	23.83	0.20	2(1H)-Naphthalenone, octahydro-4a-methyl-7-(1-methylethyl)-, (4a.alpha.,7.beta.,8a.beta.)-
94	23.90	0.24	7,10-Hexadecadienoic acid, methyl ester
95	24.00	0.20	1,3,12-Nonadecatriene
96	24.08	0.17	9-Octadecynoic acid, methyl ester
97	24.22	0.38	Cyclohexene, 1-pentyl-4-(4-propylcyclohexyl)-
98	24.34	0.19	(1S,15S)-Bicyclo[13.1.0]hexadecan-2-one
99	24.42	0.35	(Z)-3-(pentadec-8-en-1-yl)phenol
100	24.52	0.40	(1S,15S)-Bicyclo[13.1.0]hexadecan-2-one
101	24.67	0.67	Hexadecanoic acid, 2-hydroxy-1-(hydroxymethyl)ethyl ester
102	24.81	0.38	Z,Z-3,13-Octadecedien-1-ol
103	24.98	0.25	2,5-Furandione, 3-dodecyl-
104	25.08	0.50	(1,5,5,8-Tetramethyl-bicyclo[4.2.1]non-9-yl)-acetic acid, methyl ester
105	25.32	0.46	1-Chloroeicosane
106	25.59	0.42	2,5-Furandione, 3-dodecyl-
107	26.05	1.38	(Z)-3-(Heptadec-10-en-1-yl)phenol
108	26.13	1.00	(Z)-3-(Heptadec-10-en-1-yl)phenol
109	26.40	0.31	(3R,5aR,9S,9aS)-2,2,5a,9-Tetramethyloctahydro-2H-3,9a-methanobenzo[b]oxepine
110	26.72	0.69	Dichloro[(trichloromethyl)thio]methanesulfenyl chloride
111	26.90	1.30	Squalene
112	27.53	0.88	9-Tricosene, (Z)-
113	27.77	0.29	1-Benzazirene-1-carboxylic acid, 2,2,5a-trimethyl-1a-[3-oxo-1-butenyl] perhydro-, methyl ester
114	27.92	0.70	.delta.-Tocopherol
115	28.14	0.16	Acetic acid, [4-(1,1-dimethylethyl)phenoxy]-, methyl ester
116	28.31	0.42	Eicosane
117	28.83	0.34	Pulegone semicarbazone
118	29.24	0.93	Octadecane

119	29.34	0.57	Pregn-5-en-3-ol, 21-bromo-20-methyl-, (3.beta.)-
120	29.61	0.53	Vitamin E
121	30.31	0.50	Fumaric acid, pent-4-en-2-yl tridecyl ester
122	31.42	0.13	2-Ethylacridine
123	31.59	0.25	Eicosane
124	31.73	0.25	2-Ethylacridine
125	32.00	0.72	.beta.-Sitosterol
126	32.20	0.21	2-Ethylacridine
127	32.35	0.14	2-Ethylacridine
128	32.57	0.19	2-Ethylacridine
129	32.70	0.11	2-Ethylacridine
130	32.82	0.12	2-Ethylacridine
131	32.97	0.17	2-Ethylacridine
132	33.41	0.16	2-Ethylacridine
133	33.79	0.14	2-Ethylacridine
134	34.04	0.12	2-Ethylacridine
135	34.20	0.19	2-Ethylacridine
136	34.56	0.05	1,4-Bis(trimethylsilyl)benzene
137	34.67	0.03	1,2-Bis(trimethylsilyl)benzene
138	34.81	0.05	1,4-Bis(trimethylsilyl)benzene
139	34.98	0.08	1,2-Bis(trimethylsilyl)benzene
140	35.13	0.15	1,2-Bis(trimethylsilyl)benzene
141	35.56	0.08	1,2-Bis(trimethylsilyl)benzene
142	35.92	0.04	1,4-Bis(trimethylsilyl)benzene
143	36.04	0.01	1,4-Bis(trimethylsilyl)benzene
144	36.09	0.02	1,4-Bis(trimethylsilyl)benzene
145	36.27	0.01	1,2-Bis(trimethylsilyl)benzene
146	36.42	0.03	1,2-Bis(trimethylsilyl)benzene
147	36.71	0.01	1,4-Bis(trimethylsilyl)benzene
148	36.80	0.01	1,4-Bis(trimethylsilyl)benzene
149	36.90	0.01	1,2-Bis(trimethylsilyl)benzene

Table S11 Py-GC-MS components of PPs-Mo/Co_3O_4 (1:1) samples at 550 ℃.

No.	Retention time (min)	Content (%)	Compounds name
1	1.16	0.02	sec-Butylamine
2	1.50	0.01	Acetic acid, hydroxy[(1-oxo-2-propenyl)amino]-
3	3.17	22.64	Acetic acid
4	4.81	7.18	Furfural
5	5.44	1.18	1,2-Ethanediol, diacetate
6	5.62	1.13	Cyclopent-4-ene-1,3-dione
7	6.16	0.89	1H-Imidazole, 4,5-dihydro-2-methyl-
8	6.42	1.42	2-Cyclopenten-1-one, 2-hydroxy-
9	6.53	0.97	1,2-Cyclopentanedione
10	6.92	2.10	Benzaldehyde
11	7.27	0.62	Phenol
12	7.46	0.19	Phenol
13	7.99	1.26	2-Cyclopenten-1-one, 2-hydroxy-3-methyl-
14	8.28	2.12	Glycerin
15	8.93	2.37	Phenol, 2-methoxy-
16	9.44	0.43	1H-Imidazole-4-carboxylic acid, methyl ester
17	9.58	0.61	Aminopyrazine
18	9.89	0.25	4H-Pyran-4-one, 2,3-dihydro-3,5-dihydroxy-6-methyl-
19	10.30	0.25	Thiophene, 2-(2-butenyl)-, (E)-
20	10.51	0.60	Creosol
21	10.77	0.50	Catechol
22	11.13	0.43	5-Hydroxymethylfurfural
23	11.56	0.10	meta-Methoxybenzenethiol
24	11.73	0.17	Phenol, 4-ethyl-2-methoxy-
25	11.82	0.30	Xanthosine
26	12.27	0.61	2-Methoxy-4-vinylphenol
27	12.74	0.29	Phenol, 2,6-dimethoxy-
28	12.83	0.29	Eugenol
29	13.47	0.91	Vanillin
30	13.98	0.08	3,5-Dimethoxy-4-hydroxytoluene
31	14.10	0.50	trans-Isoeugenol
32	14.19	0.16	Acetamide, 2-(4-hydroxy-3-methoxyphenyl)-
33	14.51	0.09	Benzo[b]thiophene, 7-ethyl-
34	14.57	0.25	Apocynin
35	14.67	0.08	4-Isopropylbenzenethiol, S-methyl-
36	14.86	0.28	.beta.-D-Glucopyranose, 1,6-anhydro-
37	14.95	0.21	5-tert-Butylpyrogallol
38	15.07	0.39	2-Propanone, 1-(4-hydroxy-3-methoxyphenyl)-
39	15.47	0.49	2,3,5,6-Tetrafluoroanisole
40	15.64	0.18	4-(1-Hydroxyallyl)-2-methoxyphenol

41	15.78	0.24	Butyrovanillone
42	15.88	0.16	Phenol, 2,6-dimethoxy-4-(2-propenyl)-
43	16.17	0.10	d-Glycero-l-gluco-heptose
44	16.52	0.63	Benzenepropanol, 4-hydroxy-3-methoxy-
45	16.63	0.37	Benzaldehyde, 4-hydroxy-3,5-dimethoxy-
46	16.79	0.84	4-((1E)-3-Hydroxy-1-propenyl)-2-methoxyphenol
47	16.94	0.28	1H-1,3-Benzimidazole-2-methanol, 5-methoxy-1-methyl-
48	17.08	0.73	(E)-2,6-Dimethoxy-4-(prop-1-en-1-yl)phenol
49	17.43	0.26	Ethanone, 1-(4-hydroxy-3,5-dimethoxyphenyl)-
50	17.58	2.53	4-((1E)-3-Hydroxy-1-propenyl)-2-methoxyphenol
51	17.72	0.63	Tetradecanoic acid
52	17.79	0.95	2-Pentanone, 1-(2,4,6-trihydroxyphenyl)
53	18.26	0.12	Tetradecane, 1-bromo-
54	18.39	0.23	2-Heptanone, 6-(3-acetyl-1-cyclopropen-1-yl)-3-hydroxy-6-methyl-, (R*,R*)-
55	18.57	0.28	2(3H)-Naphthalenone, 4,4a,5,6,7,8-hexahydro-1-methoxy-
56	18.77	0.49	Pentadecanoic acid
57	19.11	0.13	1-ETHOXY-3-METHYL-5-NITROBENZENE
58	19.21	0.39	14-Pentadecenoic acid
59	19.42	0.21	7-Hydroxy-3-(1,1-dimethylprop-2-enyl)coumarin
60	19.60	0.68	Palmitoleic acid
61	19.82	2.08	n-Hexadecanoic acid
62	20.06	0.33	trans-Sinapaldehyde
63	20.29	0.48	Butalbital
64	20.56	0.40	Cyclopentadecanone, 2-hydroxy-
65	20.76	0.34	Heptadecanoic acid
66	20.89	0.21	Spiro[4.5]decan-7-one, 1,8-dimethyl-8,9-epoxy-4-isopropyl-
67	20.98	0.15	Cyclopentane, (2-hexyloctyl)-
68	21.12	0.24	2-Methyl-Z,Z-3,13-octadecadienol
69	21.19	0.30	Brefeldin A
70	21.49	1.67	9-Octadecenoic acid, (E)-
71	21.69	0.78	Octadecanoic acid
72	21.89	0.63	2(1H)-Naphthalenone, octahydro-4a-methyl-7-(1-methylethyl)-, (4a.alpha.,7.beta.,8a.beta.)-
73	22.13	0.28	1-Oxaspiro[2.5]octane, 4,4-dimethyl-8-methylene-2-propyl-
74	22.26	0.54	9,12-Octadecadienoic acid (Z,Z)-
75	22.43	0.36	Z-9-Pentadecenol
76	22.59	0.18	1,2-Longidione
77	22.69	0.25	Z-11-Pentadecenol
78	22.75	0.17	Z-11-Pentadecenol
79	22.84	0.37	1,4,6-Trimethyl-1,2,3,3a,4,7,8,8a-octahydro-4,7-ethanoazulene
80	22.98	0.27	Perfluoronaphthalene
81	23.09	0.56	Z-11-Pentadecenol
82	23.28	0.49	2-Dodecen-1-yl(-)succinic anhydride

83	23.41	0.70	(E)-15,16-Dinorlabda-8(17),12-dien-14-al
84	23.73	0.47	Muscone
85	23.81	0.19	7,10-Hexadecadienoic acid, methyl ester
86	23.91	0.29	Z-11-Pentadecenol
87	24.00	0.21	1,3,12-Nonadecatriene
88	24.07	0.18	Z-11-Pentadecenol
89	24.21	0.36	7,10-Hexadecadienoic acid, methyl ester
90	24.34	0.27	Z-11-Pentadecenol
91	24.42	0.33	(Z)-3-(pentadec-8-en-1-yl)phenol
92	24.54	0.47	Tricyclo[6.6.0.0(3,6)]tetradeca-1(8),4,11-triene
93	24.67	0.75	Hexadecanoic acid, 2-hydroxy-1-(hydroxymethyl)ethyl ester
94	24.81	0.45	Z,Z-3,13-Octadecedien-1-ol
95	24.96	0.25	Z-11-Pentadecenol
96	25.31	0.44	13-Octadecenal, (Z)-
97	25.52	0.17	n-Propyl 9-tetradecenoate
98	25.65	0.48	2-Dodecen-1-yl(-)succinic anhydride
99	25.89	0.56	1,2,4-Triazolo[4,3-b]pyridazine, 6,8-diphenyl-
100	26.05	1.22	(Z)-3-(Heptadec-10-en-1-yl)phenol
101	26.12	1.27	(Z)-3-(Heptadec-10-en-1-yl)phenol
102	26.39	0.38	n-Propyl 9-octadecenoate
103	26.90	2.11	Squalene
104	27.27	0.36	1-Benzazirene-1-carboxylic acid, 2,2,5a-trimethyl-1a-[3-oxo-1-butenyl] perhydro-, methyl ester
105	27.37	0.39	1-Benzazirene-1-carboxylic acid, 2,2,5a-trimethyl-1a-[3-oxo-1-butenyl] perhydro-, methyl ester
106	27.52	0.87	Cyclopentadecane
107	27.64	0.22	1-Benzazirene-1-carboxylic acid, 2,2,5a-trimethyl-1a-[3-oxo-1-butenyl] perhydro-, methyl ester
108	27.91	1.02	.delta.-Tocopherol
109	28.31	0.64	2-Ethylacridine
110	28.39	0.26	2-Ethylacridine
111	28.52	0.60	2-Ethylacridine
112	28.83	0.57	Pulegone semicarbazone
113	29.23	1.05	Eicosane
114	29.46	0.37	2-Ethylacridine
115	29.61	1.55	Vitamin E
116	30.52	0.23	Z-10-Pentadecen-1-ol acetate
117	30.60	0.28	Z-10-Pentadecen-1-ol acetate
118	31.16	0.78	2-Ethylacridine
119	31.59	0.36	Eicosane
120	31.72	0.40	2-Ethylacridine
121	31.99	1.04	.gamma.-Sitosterol
122	32.19	0.33	2-Ethylacridine
123	32.37	0.24	2-Ethylacridine

124	32.57	0.24	1,2-Bis(trimethylsilyl)benzene
125	32.70	0.18	1,2-Bis(trimethylsilyl)benzene
126	32.80	0.16	1,2-Bis(trimethylsilyl)benzene
127	32.97	0.30	1,2-Bis(trimethylsilyl)benzene
128	33.12	0.36	1,2-Bis(trimethylsilyl)benzene
129	33.41	0.22	1,2-Bis(trimethylsilyl)benzene
130	33.57	0.16	1,2-Bis(trimethylsilyl)benzene
131	33.79	0.20	1,2-Bis(trimethylsilyl)benzene
132	34.03	0.12	1,2-Bis(trimethylsilyl)benzene
133	34.18	0.15	1,2-Bis(trimethylsilyl)benzene
134	34.37	0.13	1,2-Bis(trimethylsilyl)benzene
135	34.57	0.11	1,4-Bis(trimethylsilyl)benzene
136	34.77	0.06	1,4-Bis(trimethylsilyl)benzene
137	34.99	0.12	1,4-Bis(trimethylsilyl)benzene
138	35.10	0.17	1,2-Bis(trimethylsilyl)benzene
139	35.56	0.11	1,4-Bis(trimethylsilyl)benzene
140	35.78	0.02	1,2-Bis(trimethylsilyl)benzene
141	35.93	0.07	1,2-Bis(trimethylsilyl)benzene
142	36.19	0.02	1,2-Bis(trimethylsilyl)benzene
143	36.40	0.02	1,4-Bis(trimethylsilyl)benzene
144	36.61	0.01	1,4-Bis(trimethylsilyl)benzene
145	36.73	0.00	1,2-Bis(trimethylsilyl)benzene
146	36.81	0.00	1,2-Bis(trimethylsilyl)benzene
147	36.88	0.00	1,2-Bis(trimethylsilyl)benzene
148	36.93	0.01	1,2-Bis(trimethylsilyl)benzene

Table S12 Py-GC-MS components of non-catalytic PPs samples at 700 °C.

No.	Retention time (min)	Content (%)	Compounds name
1	3.70	0.93	Toluene
2	4.11	0.36	Cyclohexan-1,4,5-triol-3-one-1-carboxylic acid
3	4.30	0.34	Acetic acid
4	5.11	0.62	Acetic acid
5	5.34	1.77	Acetic acid
6	6.25	1.80	2-Cyclopenten-1-one
7	6.91	1.15	Benzaldehyde
8	7.35	2.78	3-Furanmethanol
9	7.83	1.71	Formic acid phenyl ester
10	8.26	2.32	Benzaldehyde, 2-hydroxy-
11	8.58	1.41	1,2-Cyclopentanedione, 3-methyl-
12	9.02	3.65	Phenol, 2-methoxy-
13	9.23	2.14	Phenol, 2-methoxy-
14	9.95	0.78	Phenol, 2,5-dimethyl-
15	10.07	1.22	Phenol, 2,4-dimethyl-
16	10.22	0.28	Benzaldehyde, 3-methoxy-
17	10.37	0.78	Phenol, 2-ethyl-
18	10.59	1.84	Creosol
19	10.67	1.09	Creosol
20	11.13	4.30	Catechol
21	11.47	1.31	Phenol, 2,3,5,6-tetramethyl-
22	11.83	3.04	Phenol, 4-ethyl-2-methoxy-
23	12.30	4.64	2-Methoxy-4-vinylphenol
24	12.92	2.13	Phenol, 2-methoxy-3-(2-propenyl)-
25	13.58	2.58	1-(2,4-Dimethyl-furan-3-yl)-ethanone
26	13.80	2.34	Vanillin
27	14.17	3.07	trans-Isoeugenol
28	14.42	1.28	1-(1-Cyanocyclopentyl)pyrrolidine
29	14.88	1.86	Apocynin
30	15.10	0.79	2,5-Dimethoxybenzoic acid
31	15.32	1.92	2-Propanone, 1-(4-hydroxy-3-methoxyphenyl)-
32	15.61	0.80	4-Methyl-2,5-dimethoxybenzaldehyde
33	16.74	2.69	Benzenepropanol, 4-hydroxy-3-methoxy-
34	17.07	1.02	4-((1E)-3-Hydroxy-1-propenyl)-2-methoxyphenol
35	17.19	2.24	(E)-2,6-Dimethoxy-4-(prop-1-en-1-yl)phenol
36	17.81	3.33	Coniferyl aldehyde
37	19.39	0.66	Azoxybenzene
38	19.64	0.60	10-Methyl-9-oxabicyclo[6.4.0]dodecan-1(8)-ene
39	19.88	1.37	n-Hexadecanoic acid
40	20.11	0.47	1,7-Dimethyldibenzothiophene

41	20.23	0.88	2,5-Dimethoxycinnamic acid
42	20.53	0.34	Phenylethylene, 3'-methoxy-2,2'-dinitro-
43	20.62	0.27	1H-Pyrimidin-2-one, 4-(N'-pyridin-3-ylmethylenehydrazino)-
44	20.77	0.52	2-Cyclopenten-1-one, 4-hydroxy-2-methyl-3-phenyl-
45	21.53	1.52	9,12,15-Octadecatrienoic acid, (Z,Z,Z)-
46	21.72	0.54	Octadecanoic acid
47	21.92	0.29	[1,2,3,4]Tetrazolo[1,5-b][1,2,4]triazin-7(8H)-one, 6-(4-chlorophenyl)-
48	22.08	0.45	Benzene, 1-ethyl-4-iodo-
49	22.27	0.52	[1,2,3,4]Tetrazolo[1,5-b][1,2,4]triazin-7(8H)-one, 6-(4-chlorophenyl)-
50	22.41	0.45	4,4'-Thiobis(2-methylphenol)
51	22.58	0.34	1,3-Diamino-5,6-dihydro-7-methoxybenzo[f]quinazoline
52	22.70	0.27	1-Oxa-2-sila-5-boracyclopent-3-ene, 4,5-diethyl-2,2-dimethyl -3-(1-methylethenyl)-
53	22.83	0.67	3-Tridecylphenol
54	23.03	0.34	4H-1-Benzopyran-4-one, 2-(2-naphthalenyl)-
55	23.15	0.62	2-Chloro-7-methoxyphenazine 5-oxide
56	23.31	0.48	Phenol, 3-(dimethylamino)-
57	23.44	0.44	1-(4-Acetoxy-3-methoxyphenyl)-2-propanone
58	23.59	0.33	Phenindamine N-oxide
59	23.69	0.38	2(1H)-Phenanthrenone, 3,4,4a,4b,5,6,7,8,10,10a-decahydro-1,1,4a,7,7-pentamethyl-, [4aR-(4a.alpha.,4b.beta.,10a.beta.)]-
60	23.91	0.57	Phenol, 4-[[2-(3,4-dimethoxyphenyl)ethylamino] methyl]-2-methoxy-
61	24.00	0.23	trans-2-Undecen-1-ol
62	24.20	0.51	Phenanthrene, 7-ethenyl-1,2,3,4,4a,4b,5,6,7,9,10,10a-dodecahydro-1,1,4a,7-tetra methyl-, [4aS-(4a.alpha.,4b.beta.,7.beta.,10a.beta.)]-
63	24.26	0.23	3-Hydroxycarbofuran
64	24.41	0.42	(Z)-3-(Heptadec-10-en-1-yl)phenol
65	24.53	0.52	Eicosane
66	24.67	0.63	Hexadecanoic acid, 2-hydroxy-1-(hydroxymethyl)ethyl ester
67	24.81	0.25	1,19-Eicosadiene
68	24.89	0.25	N'-(2,4,6(1H,3H,5H)-Trioxopyrimidin-5-ylidenemethyl)-2-nitrobe nzhydrazide
69	24.98	0.28	2-Nitrophenyl cinnamamide
70	25.05	0.82	3-phenylprop-2-enoic anhydride
71	25.31	0.62	Heneicosane
72	25.45	0.29	D-Homoestra-1,3,5(10),8-tetraen-3,17-diol
73	25.53	0.28	1,1'-Bi-2-naphthol
74	25.60	0.62	2-Quinoxalinecarboxamide, 3-amino-6,7-difluoro-, 1,4-dioxide
75	25.92	1.23	1,1'-Biphenyl-3,4,4'-trimethoxy-6'-formyl-

76	26.06	1.16	Hexadecane
77	26.52	0.27	7-Methoxy-3-(3,4-dimethoxyphenyl)-4H-chromen-4-one
78	26.59	0.36	4,6-Androstadien-17.beta.-ol-3-one
79	26.78	0.54	1-Chloroeicosane
80	26.89	0.74	Squalene
81	27.12	0.38	Estra-1,3,5(10)-trien-17-one, 3-hydroxy-2-methoxy-
82	27.26	0.72	4,9(11)-Androstadiene-3,17-dione
83	27.51	0.60	Cyclopentadecane
84	27.63	0.26	Cyclopenta[c]furo[3',2':4,5]furo[2,3-h][1]benzopyran-1,11-dione, 2,3,6a,9a-tetrahydro-3-hydroxy-4-methoxy-, [3S-(3.alpha.,6a.alpha.,9a.alpha.)]-
85	27.78	0.86	10,11-Dihydro-10-hydroxy-2,3,6-trimethoxydibenz(b,f)oxepin
86	28.01	0.26	2,5-Cyclohexadiene-1,4-dione, 2,5-dihydroxy-3,6-bis(4-hydroxyphenyl)-
87	28.29	0.66	Docosane
88	28.49	0.63	8-Methyl-2-phenylquinoline-6-.alpha.-pyridylmethanol
89	28.74	0.42	N-Benzo[1,3]dioxol-5-yl-2-(3H-imidazo[4,5-b]pyridin-2-ylsulfanyl)-acetamide
90	29.32	0.51	Stigmastan-3,5-diene
91	29.54	1.07	Ethanone, 2-(1H-imidazo[4,5-b]pyridin-2-yl)-1-(4-morpholyl)-
92	30.26	0.20	.alpha.,3'-Bicinnamaldehyde, 4,4'-dihydroxy-3,5'-dimethoxy-
93	30.44	0.17	4-(Diethoxyphosphinoyl)-N-(prop-2'-enyl)butanamide
94	30.83	0.47	2(3H)-Benzofuranone, 3a,4,5,7a-tetrahydro-3a, 6-dimethyl-, cis-(.+-.)-
95	31.16	0.43	Stigmasterol
96	31.48	0.20	2-Butenenitrile, 2-chloro-3-(4-methoxyphenyl)-
97	31.72	0.21	Nonacos-1-ene
98	32.02	0.55	.gamma.-Sitosterol
99	32.18	0.15	19-Norethindrone, O-methyloxime
100	32.38	0.12	2-Butenenitrile, 2-chloro-3-(4-methoxyphenyl)-
101	32.57	0.08	2H-3,9a-Methano-1-benzoxepin, octahydro-2,2,5a,9-tetramethyl-, [3R-(3.alpha.,5a.alpha.,9.alpha.,9a.alpha.)]-
102	32.69	0.07	7-Isopropenyl-1,4a-dimethyl-4,4a,5,6,7,8-hexahydro-3H-naphthalen-2-one
103	32.80	0.21	19-Norethindrone, O-methyloxime
104	33.57	0.08	19-Norethindrone, O-methyloxime
105	33.77	0.05	19-Norethindrone, O-methyloxime
106	33.88	0.05	19-Norethindrone, O-methyloxime
107	34.02	0.07	2-(Heneicosa-3,6,9,12,15,18-hexaenyl)-N-methylimidazoline
108	34.21	0.13	2H-3,9a-Methano-1-benzoxepin, octahydro-2,2,5a,9-tetramethyl-, [3R-(3.alpha.,5a.alpha.,9.alpha.,9a.alpha.)]-
109	34.58	0.05	2H-3,9a-Methano-1-benzoxepin, octahydro-2,2,5a,9-tetramethyl-, [3R-(3.alpha.,5a.alpha.,9.alpha.,9a.alpha.)]-
110	34.73	0.02	2-Bromo-4,5-dimethoxycinnamic acid

111	34.91	0.05	2-Butenenitrile, 2-chloro-3-(4-methoxyphenyl)-
112	35.25	0.27	Nonacos-1-ene
113	35.73	0.03	5H-dibenzo[a,d]cyclohepten-5-amine
114	35.91	0.01	5H-dibenzo[a,d]cyclohepten-5-amine
115	35.99	0.01	5H-dibenzo[a,d]cyclohepten-5-amine
116	36.20	0.04	1-Hexacosene
117	36.40	0.01	19-Norethindrone, O-methyloxime
118	36.75	0.01	1-Oxa-2-sila-5-boracyclopent-3-ene, 4,5-diethyl-2,2-dimethyl-3-(1-methylethenyl)-
119	36.90	0.00	5H-dibenzo[a,d]cyclohepten-5-amine

Table S13 Py-GC-MS components of PPs-Co_3O_4 samples at 700 °C.

No.	Retention time (min)	Content (%)	Compounds name
1	3.75	3.96	Acetic acid
2	4.15	3.08	Acetic acid
3	4.73	1.70	Furfural
4	4.87	2.59	1H-Imidazole, 1,5-dimethyl-
5	5.27	1.94	Furfural
6	5.54	1.83	2-Cyclopenten-1-one
7	5.64	1.12	Bicyclo[4.2.0]octa-1,3,5-triene
8	6.79	3.14	Cyclohexanone
9	7.18	1.02	2-Furancarboxaldehyde, 5-methyl-
10	7.25	0.70	1,4-Pentadiene
11	7.45	1.65	Phenol
12	7.82	1.68	1,4-Butanediamine, 2,3-dimethoxy-N,N,N',N'-tetramethyl-, [S-(R*,R*)]-
13	8.22	1.82	1,2-Cyclopentanedione, 3-methyl-
14	8.36	1.00	Phenol, 2-methyl-
15	8.53	0.84	Phenol, 2-methyl-
16	8.75	0.69	Phenol, 3-methyl-
17	8.88	1.55	Phenol, 2-methoxy-
18	9.05	1.52	Phenol, 2-methoxy-
19	9.52	0.97	3-Hydroxypyridine monoacetate
20	9.85	0.53	Phenol, 2,4-dimethyl-
21	9.92	0.55	Phenol, 2,3-dimethyl-
22	10.03	0.37	2-Hydroxy-5-methylbenzaldehyde
23	10.12	0.46	Benzaldehyde, 3-methoxy-
24	10.19	0.52	Phenol, 3-ethyl-
25	10.27	0.82	2,3-Dihydroxybenzaldehyde
26	10.48	0.98	Creosol
27	10.56	0.72	Creosol
28	10.90	5.32	Catechol
29	11.22	1.67	Phenol, 4-ethyl-3-methyl-
30	11.70	2.66	1,2-Benzenediol, 3-methyl-
31	12.01	0.91	2-Allylphenol
32	12.17	2.93	1,2-Benzenediol, 4-methyl-
33	12.32	1.90	2-Methoxy-4-vinylphenol
34	12.73	1.47	Phenol, 4-(2-propenyl)-
35	12.86	0.97	Eugenol
36	13.01	1.62	1,5,6,7-Tetramethylbicyclo[3.2.0]hepta-2,6-diene
37	13.17	0.58	2-Allyl-4-methylphenol
38	13.38	2.72	4-Ethylcatechol
39	13.55	0.66	Phenol, 2-methoxy-4-(1-propenyl)-, (Z)-

40	13.66	2.09	Vanillin
41	13.88	1.04	2-Ethyl-3-methoxypyrazine
42	14.14	2.92	trans-Isoeugenol
43	14.30	1.20	Benzenamine, 5-methoxy-2-methyl-
44	14.65	0.46	Benzene, 3-ethyl-1,2,4,5-tetramethyl-
45	14.74	0.97	Apocynin
46	15.32	0.39	Ethyl (Z)-cinnamate
47	15.54	0.85	4-Methyl-2,5-dimethoxybenzaldehyde
48	16.08	0.34	Ethanone, 1-phenyl-, oxime
49	16.51	0.29	Phenol, 2,6-dimethoxy-4-(2-propenyl)-
50	16.62	0.82	Benzenepropanol, 4-hydroxy-3-methoxy-
51	16.73	0.64	2,3-Dimethoxybenzoic acid
52	16.89	1.53	4-((1E)-3-Hydroxy-1-propenyl)-2-methoxyphenol
53	17.13	1.48	Phenol, 2,6-dimethoxy-4-(2-propenyl)-
54	17.43	0.41	3,4,5-Trimethyl-1H-pyrano[2,3-c]pyrazol-6-one
55	17.49	0.38	3-Methyl-6-nitrobenzoic acid
56	17.68	2.52	Coniferyl aldehyde
57	17.88	0.43	1-(1-Hydroxybutyl)-2,5-dimethoxybenzene
58	18.33	0.42	2,3,5,6-Tetramethylterephthalaldehyde
59	18.63	0.19	Chalcone
60	19.11	0.25	Spirafolide
61	19.42	0.37	3-Hydroxy-4-nitrobenzaldehyde
62	19.58	0.38	Cyclohexane, 1,2,3,4-bis(epoxy)-2,6,6-trimethyl-1-(pent-2-en-4-one-2-yl)-
63	19.67	0.16	4-Nitro-5,6,7,8-tetrahydronaphthalen-1-ol
64	19.82	1.11	n-Hexadecanoic acid
65	20.10	0.63	5-benzofuranol, 2-ethoxy-2,3-dihydro-3,3-dimethyl-
66	20.30	0.41	Benzene, 2-methoxy-1-methyl-3,5-dinitro-
67	20.54	0.36	[1,2,3,4]Tetrazolo[1,5-b][1,2,4]triazin-7(8H)-one, 6-(4-chlorophenyl)-
68	20.73	0.40	Heptadecanoic acid
69	20.87	0.29	Benzene, 1-bromo-4-(ethoxymethyl)-
70	21.01	0.17	10-Heptadecen-8-ynoic acid, methyl ester, (E)-
71	21.06	0.23	4-Oxatricyclo[4.3.1.1(3,8)]undecane
72	21.22	0.28	2,3,4-Trimethoxybenzoic acid
73	21.34	0.12	Spiro[bicyclo[6.1.0]nonane-9,1'-cyclopentane], 3'-methylene-
74	21.48	0.58	9,12,15-Octadecatrienoic acid, (Z,Z,Z)-
75	21.57	0.17	Naphthalene, 1,2,3,4,4a,5,6,8a-octahydro-4a,8-dimethyl-2-(1-methylethenyl)-, [2R-(2.alpha.,4a.alpha.,8a.beta.)]-
76	21.68	0.53	Octadecanoic acid
77	21.87	0.13	[1,2,3,4]Tetrazolo[1,5-b][1,2,4]triazin-7(8H)-one, 6-(4-chlorophenyl)-
78	22.13	0.22	5-Chloro-1,10-phenanthroline

79	22.44	0.27	2-Cyclohexen-1-one, 3-(3-hydroxybutyl)-2,4,4-trimethyl-
80	22.58	0.19	Azacyclotridecan-2-one, 1-(3-aminopropyl)-
81	22.72	0.23	Benzene, 1,1'-[1-(2-propenyl)-1,2-ethanediyl]bis-
82	22.81	0.23	Oxalic acid, hexyl 2-methylphenyl ester
83	22.97	0.20	Estr-4-ene-3,17-dione
84	23.13	0.40	2-Propenoic acid, 3-phenyl-, 4-methylphenyl ester
85	23.41	0.26	Eicosanoic acid
86	23.52	0.13	Cyanoacetic acid, pentadecyl ester
87	23.60	0.18	1-Octadecene
88	23.67	0.39	1-Octadecene
89	23.98	0.20	Henicosanal
90	24.15	0.27	Propenone, 3-(4-chlorophenyl)-1-(2-methylphenyl)-
91	24.24	0.22	Pentadecanoic acid
92	24.39	0.21	1-(5,6-Dimethyl-2-pyrazinyl)-3-methyl-1-butanone
93	24.52	0.23	Octadecane
94	24.68	0.30	(E)-Labda-8(17),12-diene-15,16-dial
95	24.79	0.23	Oxirane, tridecyl-
96	24.91	0.45	Silane, ethoxytriethyl-
97	25.09	0.16	Estra-1,3,5(10)-triene-7,17-dione, 3-hydroxy-
98	25.29	0.29	Hexadecane
99	25.40	0.14	Dibenz[a,c]cyclohexane, 2,4,7-trimethoxy-
100	25.48	0.14	5-Chlorovaleric acid, octadecyl ester
101	25.57	0.22	Oxirane, tridecyl-
102	25.81	0.31	9,10-Anthracenedione, 1,2,5,8-tetrahydroxy-
103	25.87	0.26	3-(4-Chlorophenyl)-9-fluoro-[1,2,4]triazolo[4,3-c]quinazoline
104	26.04	0.25	Hexadecane
105	26.16	0.12	19-Norethindrone, O-methyloxime
106	26.23	0.11	5-Chlorovaleric acid, octadecyl ester
107	26.32	0.16	2-Methyloctadecanedi-7,8-ol, 7-O-trifluoroacetyl-
108	26.48	0.21	7-Methoxy-3-(3,4-dimethoxyphenyl)-4H-chromen-4-one
109	26.55	0.14	benzene, 1,1'-(4,6-decadiyne-1,10-diyl)bis-
110	26.65	0.10	5-Chlorovaleric acid, octadecyl ester
111	26.76	0.19	Docosane
112	26.88	0.20	Squalene
113	27.01	0.41	Benzenamine, 2-ethyl-N-(4,4-dimethylthiazolidin-2-ylidene)-
114	27.37	0.16	4-Nitrobenzoic acid, 10-undecenyl ester
115	27.48	0.30	Eicosane
116	27.79	0.36	2-Butenenitrile, 2-chloro-3-(4-methoxyphenyl)-
117	27.95	0.09	2-Butenenitrile, 2-chloro-3-(4-methoxyphenyl)-
118	28.09	0.12	2-Butenenitrile, 2-chloro-3-(4-methoxyphenyl)-
119	28.49	0.32	Cholesta-3,5-diene
120	28.73	0.20	2H-3,9a-Methano-1-benzoxepin, octahydro-2,2,5a,9-tetramethyl-, [3R-(3.alpha.,5a.alpha.,9.alpha.,9a.alpha.)]-
121	28.85	0.09	5-Hexenoic acid, 6-[p-chlorophenyl]-2,4-dioxo-, ethyl ester

122	28.96	0.11	Stigmasta-5,22-dien-3-ol, acetate, (3.beta.)-
123	29.09	0.14	2H-3,9a-Methano-1-benzoxepin, octahydro-2,2,5a,9-tetramethyl-, [3R-(3.alpha.,5a.alpha.,9.alpha.,9a.alpha.)]-
124	29.30	0.29	Stigmastan-3,5-diene
125	29.45	0.08	2-Bromo-4,5-dimethoxycinnamic acid
126	29.56	0.09	5H-dibenzo[a,d]cyclohepten-5-amine
127	29.66	0.26	5H-dibenzo[a,d]cyclohepten-5-amine
128	29.97	0.15	2-Bromo-4,5-dimethoxycinnamic acid
129	30.26	0.35	5,5'-Di(ethoxycarbonyl)-3,3'-dimethyl-4,4'-dipropyl-2,2'-dipyrryl methane
130	30.79	0.18	2-Bromo-4,5-dimethoxycinnamic acid
131	31.10	0.28	5,5'-Di(ethoxycarbonyl)-3,3'-dimethyl-4,4'-dipropyl-2,2'-dipyrryl methane
132	31.58	0.20	2-Bromo-4,5-dimethoxycinnamic acid
133	31.92	0.10	.beta.-Sitosterol
134	32.14	0.20	2-Bromo-4,5-dimethoxycinnamic acid
135	32.45	0.06	2-Bromo-4,5-dimethoxycinnamic acid
136	32.61	0.05	2-Bromo-4,5-dimethoxycinnamic acid
137	32.74	0.14	2-(Acetoxymethyl)-3-(methoxycarbonyl)biphenylene
138	33.78	0.07	2-(Acetoxymethyl)-3-(methoxycarbonyl)biphenylene
139	34.17	0.04	2-(Acetoxymethyl)-3-(methoxycarbonyl)biphenylene
140	34.33	0.02	2-(Acetoxymethyl)-3-(methoxycarbonyl)biphenylene
141	34.41	0.07	2-(Acetoxymethyl)-3-(methoxycarbonyl)biphenylene
142	34.87	0.05	Adamantane-1-(3,3-dichloropropyn-1-yl)
143	35.05	0.01	2-(Acetoxymethyl)-3-(methoxycarbonyl)biphenylene
144	35.12	0.04	2-Bromo-4,5-dimethoxycinnamic acid
145	35.98	0.01	Adamantane-1-(3,3-dichloropropyn-1-yl)
146	36.06	0.01	Adamantane-1-(3,3-dichloropropyn-1-yl)
147	36.18	0.02	Adamantane-1-(3,3-dichloropropyn-1-yl)
148	36.77	0.00	Adamantane-1-(3,3-dichloropropyn-1-yl)

Table S14 Py-GC-MS components of PPs-Co_3O_4 samples at 700 °C.

No.	Retention time (min)	Content (%)	Compounds name
1	2.95	8.14	Acetic acid
2	3.22	9.15	Acetic acid
3	4.67	1.21	Furfural
4	4.86	5.00	Furfural
5	5.06	2.58	Furfural
6	5.50	1.46	2-Propanone, 1-(acetyloxy)-
7	5.65	2.40	Bicyclo[4.2.0]octa-1,3,5-triene
8	6.09	0.64	3-Heptyne
9	6.19	1.13	3-Hexene, (E)-
10	6.42	1.15	2-Cyclopenten-1-one, 2-hydroxy-
11	6.54	0.43	Cyclohexanone
12	6.67	0.42	Cyclohexanone
13	6.93	1.41	Benzaldehyde
14	7.00	0.48	Benzaldehyde
15	7.08	0.56	Bicyclo[3.1.0]hexan-2-one
16	7.23	1.01	Phenol
17	7.60	0.93	1,4-Butanediamine, 2,3-dimethoxy-N,N,N',N'-tetramethyl-, [S-(R*,R*)]-
18	7.99	0.97	1,2-Cyclopentanedione, 3-methyl-
19	8.29	0.99	Benzaldehyde, 2-hydroxy-
20	8.38	1.12	Phenol, 2-methyl-
21	8.72	1.65	Phenol, 3-methyl-
22	8.92	2.13	Phenol, 2-methoxy-
23	9.24	0.32	Phenol, 2,6-dimethyl-
24	9.38	0.79	3-Hydroxypyridine monoacetate
25	9.85	0.70	Phenol, 2,4-dimethyl-
26	10.09	0.76	Phenol, 2-propyl-
27	10.17	0.36	Benzoic acid
28	10.28	0.22	Creosol
29	10.49	1.03	Creosol
30	10.61	1.97	Catechol
31	10.89	0.66	Benzofuran, 2,3-dihydro-
32	11.00	0.36	1,3-Cyclohexadiene, 1-methyl-4-(1-methylethyl)-
33	11.07	0.47	2-Isopropoxyphenol
34	11.15	0.78	5-Hydroxymethylfurfural
35	11.49	1.05	1,2-Benzenediol, 3-methyl-
36	11.72	0.61	Phenol, 4-ethyl-2-methoxy-
37	11.78	0.59	Hydroquinone
38	11.93	2.11	1,2-Benzenediol, 4-methyl-
39	12.24	0.92	2-Methoxy-4-vinylphenol

40	12.49	0.34	1,2-Benzenediol, 3-methyl-
41	12.63	0.51	Phenol, 4-(2-propenyl)-
42	12.73	0.43	Phenol, 2,6-dimethoxy-
43	12.80	0.46	Eugenol
44	12.88	0.35	1H-Indenol
45	12.95	0.43	Benzaldehyde, 4-(1-methylethyl)-
46	13.19	1.99	4-Ethylcatechol
47	13.47	1.23	Vanillin
48	13.65	1.05	2-Allyl-4-methylphenol
49	14.08	0.75	Phenol, 2-methoxy-4-(1-propenyl)-
50	14.17	0.49	Pyrrolidine, 1-(1-cyclopenten-1-yl)-
51	14.59	0.69	Apocynin
52	15.52	2.52	trans-3-(2-Nitrovinyl)pyridine
53	15.64	0.40	1,3-Benzenedicarboxylic acid, 4-methyl-
54	15.81	1.00	Glycine, N-ethoxycarbonyl-, octyl ester
55	16.08	0.85	7-Benzofuranol, 2,3-dihydro-2,2-dimethyl-
56	16.50	0.94	Benzenepropanol, 4-hydroxy-3-methoxy-
57	16.61	0.62	2,3-Dimethoxybenzoic acid
58	16.77	0.92	4-((1E)-3-Hydroxy-1-propenyl)-2-methoxyphenol
59	17.06	0.87	(E)-2,6-Dimethoxy-4-(prop-1-en-1-yl)phenol
60	17.57	2.49	4-((1E)-3-Hydroxy-1-propenyl)-2-methoxyphenol
61	17.79	0.32	1-(1-Hydroxybutyl)-2,5-dimethoxybenzene
62	18.25	0.24	4-Methoxy-7-methylindan-1-one
63	18.43	0.40	Propanehydrazide, N2-(4-methoxyphenyl)-
64	18.58	0.33	Benzoic acid, 2-methyl-3-nitro-
65	18.71	0.15	Pentadecanoic acid
66	18.79	0.23	4-Nitro-5,6,7,8-tetrahydronaphthalen-1-ol
67	18.90	0.27	Indeno[1,2-b]pyridin-5-ol, 7-amino-
68	19.15	0.44	4-((1E)-3-Hydroxy-1-propenyl)-2-methoxyphenol
69	19.32	0.30	Phenol, 4-methyl-2,6-dinitro-
70	19.62	0.11	3-Methyl-6-nitrobenzoic acid
71	19.78	1.55	n-Hexadecanoic acid
72	20.03	0.52	2,5-Dimethoxycinnamic acid
73	20.25	0.35	Benzene, 2-methoxy-1-methyl-3,5-dinitro-
74	20.50	0.27	Benzene, 2-methoxy-1-methyl-3,5-dinitro-
75	20.60	0.09	Benzoic acid, 3-methyl-2-nitro-
76	20.71	0.27	Heptadecanoic acid
77	20.87	0.23	Benzene, 4-methyl-1,2-dinitro-
78	20.95	0.20	Benzenemethanol, 3-phenoxy-
79	21.05	0.18	Methyl tetradec-5-ynoate
80	21.32	0.13	4-Oxatricyclo[4.3.1.1(3,8)]undecane
81	21.45	0.74	(Z)6,(Z)9-Pentadecadien-1-ol
82	21.66	0.49	Octadecanoic acid
83	21.85	0.10	9-Borabicyclo[3.3.1]nonane, 9-(1,2-diphenylethyl)-

84	21.93	0.31	Benzenamine, 2-chloro-N,N-diethyl-4-nitro-
85	22.09	0.21	1,1'-Biphenyl, 2,2'-dimethoxy-
86	22.19	0.09	Benzaldehyde, 5-bromo-2-methoxy-
87	22.55	0.11	Benzene, 1-bromo-4-(ethoxymethyl)-
88	22.61	0.12	[1,2,3,4]Tetrazolo[1,5-b][1,2,4]triazin-7(8H)-one, 6-(4-chlorophenyl)-
89	22.80	0.24	Oxalic acid, hexyl 2-methylphenyl ester
90	22.89	0.09	Methane, bis(p-methoxyphenyl)-,
91	22.95	0.16	2,4-Diamino-5-[3,4-propylidenedioxybenzyl]pyrimidine
92	23.13	0.41	11-Dodecen-1-ol difluoroacetate
93	23.27	0.31	Phenanthrene, 7-ethenyl-1,2,3,4,4a,4b,5,6,7,9,10,10a -dodecahydro-1,1,4a,7-tetramethyl-, [4aS-(4a.alpha., 4b. beta.,7.beta.,10a.beta.)]-
94	23.40	0.24	Eicosanoic acid
95	23.70	0.49	Styrene, 2,3,5,6-tetraethyl-4-vinyl-
96	23.87	0.18	Diphenylketone-3-methoxy-4-carboxylic acid, ethyl(ester)
97	23.98	0.24	Oxirane, hexadecyl-
98	24.09	0.15	N-[3,4-(Methylenedioxy)phenyl]-3-oxobutyramide
99	24.13	0.12	R (2-Amino-5-((trifluoromethyl)sulfonyl)phenyl)(hydroxy)azane oxide
100	24.38	0.26	(Z)-3-(pentadec-8-en-1-yl)phenol
101	24.52	0.28	Octadecane
102	24.63	0.30	Propanamide, N-(3-bromophenyl)-3-(1-piperidyl)-
103	24.79	0.28	Oxirane, heptadecyl-
104	24.89	0.25	2-Propenamide, N-(3-nitrophenyl)-3-phenyl-
105	25.03	0.21	(1,5,5,8-Tetramethyl-bicyclo[4.2.1]non-9-yl)-acetic acid, methyl ester
106	25.09	0.20	D-Homoestra-1,3,5(10),8-tetraen-3,17-diol
107	25.22	0.17	6-Hydroxynicotinic acid, 2TMS derivative
108	25.30	0.23	Nonadecane
109	25.39	0.17	Dibenz[a,c]cyclohexane, 2,4,7-trimethoxy-
110	25.47	0.21	Estra-1,3,5(10)-trien-17-one, 2,3-dihydroxy-
111	25.57	0.38	Oxirane, hexadecyl-
112	25.80	0.78	4-Methoxy-4',5'-methylenedioxybiphenyl-2-carboxylic acid
113	26.04	0.57	Hexadecane
114	26.24	0.12	Trinexapac-ethyl, TMS derivative
115	26.32	0.28	2-Butenenitrile, 2-chloro-3-(4-methoxyphenyl)-
116	26.47	0.14	7-Methoxy-3-(3,4-dimethoxyphenyl)-4H-chromen-4-one
117	26.55	0.20	2H-3,9a-Methano-1-benzoxepin, octahydro-2,2,5a,9-tetramethyl-, [3R-(3.alpha.,5a.alpha.,9.alpha.,9a.alpha.)]-
118	26.65	0.15	2-Butenenitrile, 2-chloro-3-(4-methoxyphenyl)-
119	26.76	0.29	Tricosane
120	26.87	0.28	Squalene
121	27.06	0.65	Aspidofractinin-3-one, 17-methoxy-, (2.alpha.,5.alpha.)-

122	27.48	0.38	Tetracosane
123	27.66	0.16	2-(Acetoxymethyl)-3-(methoxycarbonyl)biphenylene
124	27.78	0.12	2-(Acetoxymethyl)-3-(methoxycarbonyl)biphenylene
125	27.94	0.13	2-(Acetoxymethyl)-3-(methoxycarbonyl)biphenylene
126	28.09	0.27	2-(Acetoxymethyl)-3-(methoxycarbonyl)biphenylene
127	28.49	0.27	2-Bromo-4,5-dimethoxycinnamic acid
128	28.72	0.20	Adamantane-1-(3,3-dichloropropyn-1-yl)
129	28.84	0.10	Adamantane-1-(3,3-dichloropropyn-1-yl)
130	29.09	0.14	Acetic acid, [4-(1,1-dimethylethyl)phenoxy]-, methyl ester
131	29.30	0.36	Stigmastan-3,5-diene
132	29.57	0.18	Adamantane-1-(3,3-dichloropropyn-1-yl)
133	29.73	0.10	Thymol, TBDMS derivative
134	29.87	0.08	Thymol, TBDMS derivative
135	29.98	0.15	2-(n-Propyl)oxybenzylidene acetophenone
136	30.26	0.22	Adamantane-1-(3,3-dichloropropyn-1-yl)
137	30.97	0.06	2-(Acetoxymethyl)-3-(methoxycarbonyl)biphenylene
138	31.24	0.10	2-(Acetoxymethyl)-3-(methoxycarbonyl)biphenylene
139	31.43	0.04	2'-Hydroxypropiophenone, TMS derivative
140	31.53	0.07	Thymol, TBDMS derivative
141	31.69	0.11	2-(Acetoxymethyl)-3-(methoxycarbonyl)biphenylene
142	31.92	0.48	.beta.-Sitosterol
143	32.49	0.06	2'-Hydroxypropiophenone, TMS derivative
144	32.62	0.06	2'-Hydroxypropiophenone, TMS derivative
145	33.17	0.11	2-Ethylacridine
146	33.26	0.14	Thymol, TMS derivative
147	33.70	0.06	Thymol, TMS derivative
148	34.57	0.02	Thymol, TMS derivative
149	35.16	0.04	2-Ethylacridine
150	35.29	0.01	Thymol, TMS derivative
151	35.74	0.01	Thymol, TMS derivative
152	35.89	0.01	Thymol, TMS derivative
153	36.81	0.00	Thymol, TMS derivative

Table S15 Py-GC-MS components of PPs -Mo/Co_3O_4 (1:1) samples at 700 °C.

No.	Retention time (min)	Content (%)	Compounds name
1	3.74	1.13	Toluene
2	4.27	1.50	Acetic acid
3	4.39	0.95	Acetic acid
4	4.67	2.34	Acetic acid
5	4.81	1.10	Furfural
6	4.93	1.65	Furfural
7	5.11	1.29	Furfural
8	5.83	1.80	Furfural
9	6.13	2.62	2-Propanone, 1-(acetyloxy)-
10	6.60	0.79	4-Octyne
11	6.87	0.89	Benzaldehyde
12	6.96	2.30	Cyclohexanone
13	7.59	1.20	Formic acid phenyl ester
14	7.93	1.97	2H-Pyran, 3,4-dihydro-2-methoxy-
15	8.24	0.99	Benzaldehyde, 2-hydroxy-
16	8.39	1.81	Phenol, 3-methyl-
17	8.62	0.68	Phenol, 2-methyl-
18	8.78	0.51	p-Cresol
19	8.92	1.90	Phenol, 2-methoxy-
20	9.11	1.27	Phenol, 2-methoxy-
21	9.26	0.70	5-Methylbenzimidazole
22	9.92	1.22	Phenol, 2,4-dimethyl-
23	10.04	0.62	3-Hydroxy-4-methylbenzaldehyde
24	10.16	0.45	Benzaldehyde, 3-methoxy-
25	10.27	0.57	Phenol, 3-ethyl-
26	10.37	0.57	2,3-Dihydroxybenzaldehyde
27	10.54	2.03	Creosol
28	11.04	4.73	Catechol
29	11.20	0.96	Phenol, 4-ethyl-3-methyl-
30	11.36	0.39	2-Isopropoxyphenol
31	11.45	1.25	Ethanone, 1-(2-hydroxy-5-methylphenyl)-
32	11.76	3.11	O-Methoxy-.alpha.-methylbenzyl alcohol
33	12.05	0.82	2-Allylphenol
34	12.26	2.25	1,2-Benzenediol, 4-methyl-
35	12.33	1.68	2-Methoxy-4-vinylphenol
36	12.76	1.12	Phenol, 4-(2-propenyl)-
37	12.88	1.37	Eugenol
38	13.20	0.51	Benzaldehyde, 4-(1-methylethyl)-
39	13.46	2.64	1,3-Benzenediol, 4-ethyl-
40	13.57	0.69	trans-Isoeugenol

41	13.75	2.22	Benzaldehyde, 3-hydroxy-4-methoxy-
42	14.16	2.63	trans-Isoeugenol
43	14.37	1.26	2-Propanone, 1-(4-hydroxy-3-methoxyphenyl)-
44	14.67	0.48	Benzene, 1,3,5-triethyl-
45	14.81	1.16	Apocynin
46	15.05	0.63	Methiocarb-anisole
47	15.57	0.78	4-Methyl-2,5-dimethoxybenzaldehyde
48	15.92	2.45	.alpha.-Amino-3'-hydroxy-4'-methoxyacetophenone
49	16.43	1.02	1-(3,3-Dimethyl-1-yl)-2,2-dimethylcyclopropene-3-carboxylic acid
50	16.54	0.42	Phenol, 2,6-dimethoxy-4-(2-propenyl)-
51	16.68	1.08	Benzenepropanol, 4-hydroxy-3-methoxy-
52	16.78	0.86	Benzaldehyde, 4-hydroxy-3,5-dimethoxy-
53	16.95	1.45	4-((1E)-3-Hydroxy-1-propenyl)-2-methoxyphenol
54	17.16	1.91	(E)-2,6-Dimethoxy-4-(prop-1-en-1-yl)phenol
55	17.52	0.83	2-Propenoic acid, 3-(4-hydroxyphenyl)-, methyl ester
56	17.75	2.55	Coniferyl aldehyde
57	18.35	0.32	3-Buten-2-ol, 4-(2,6,6-trimethyl-1-cyclohexen-1-yl)-
58	18.85	0.32	1H-indene-4-carboxylic acid, 2,3-dihydro-7-methyl-, ethyl ester
59	19.11	0.22	3,8,11-Trioxatetracyclo[4.4.1.0(2,4).0(7,9)]undecane, (1.alpha.,2.alpha.,4.alpha.,6.alpha.,7.beta.,9.beta.)-
60	19.44	0.31	Phenol, o-2-benzimidazolyl-
61	19.61	0.40	4-Methoxy-3-nitrobenzaldehyde
62	19.84	0.98	n-Hexadecanoic acid
63	19.96	0.24	2-Decene, 2-methyl-
64	20.12	0.79	2,5-Dimethoxycinnamic acid
65	20.32	0.41	Benzoic acid, 3-methyl-2-nitro-
66	20.51	0.19	Benzene, 1-bromo-4-(ethoxymethyl)-
67	20.57	0.24	6-Ethyl-5-[4-pyridinyl]-2,4-pyrimidinediamine
68	20.74	0.43	Heptadecanoic acid
69	21.03	0.23	[1,2,3,4]Tetrazolo[1,5-b][1,2,4]triazin-7(8H)-one, 6-(4-chlorophenyl)-
70	21.11	0.22	[1,2,3,4]Tetrazolo[1,5-b][1,2,4]triazin-7(8H)-one, 6-(4-chlorophenyl)-
71	21.23	0.30	[1,2,3,4]Tetrazolo[1,5-b][1,2,4]triazin-7(8H)-one, 6-(4-chlorophenyl)-
72	21.34	0.14	5H,6H-Furo[2,3-d]pyrimidin-2-amine
73	21.49	0.57	9,12,15-Octadecatrien-1-ol, (Z,Z,Z)-
74	21.58	0.19	Ketone, methyl 2-methyl-1-cyclohexen-1-yl, semicarbazone
75	21.69	0.35	Octadecanoic acid
76	21.78	0.14	[1,2,3,4]Tetrazolo[1,5-b][1,2,4]triazin-7(8H)-one, 6-(4-chlorophenyl)-
77	21.87	0.19	[1,2,3,4]Tetrazolo[1,5-b][1,2,4]triazin-7(8H)-one, 6-(4-chlorophenyl)-

78	22.00	0.41	[1,2,3,4]Tetrazolo[1,5-b][1,2,4]triazin-7(8H)-one, 6-(4-chlorophenyl)-
79	22.16	0.20	[1,2,3,4]Tetrazolo[1,5-b][1,2,4]triazin-7(8H)-one, 6-(4-chlorophenyl)-
80	22.26	0.17	1,4-Cyclohexanedicarboxylic acid, 2,5-dioxo-, diethyl ester
81	22.34	0.12	Benzene, 1-bromo-4-(ethoxymethyl)-
82	22.45	0.33	[1,2,3,4]Tetrazolo[1,5-b][1,2,4]triazin-7(8H)-one, 6-(4-chlorophenyl)-
83	22.82	0.28	1,4,6-Trimethyl-1,2,3,3a,4,7,8,8a-octahydro-4,7-ethanoazulene
84	22.98	0.20	19-Norandrost-4-en-3,17-dione (8.beta.,9.beta.,10.alpha.)
85	23.14	0.43	2-Propenamide, N-(3-nitrophenyl)-3-phenyl-
86	23.25	0.29	(2E)-4-(4-Hydroxy-3-methoxyphenyl)-2-butanone oxime
87	23.41	0.21	Eicosanoic acid
88	23.53	0.32	[1,2,3,4]Tetrazolo[1,5-b][1,2,4]triazin-7(8H)-one, 6-(4-chlorophenyl)-
89	23.72	0.24	3-Fluorobenzaldehyde semicarbazone
90	23.89	0.31	1-Selena-2-sila-5-boracyclopent-3-ene, 4,5-diethyl-2,2-dimethyl-3-(1-methylethenyl)-
91	23.98	0.26	Henicosanal
92	24.24	0.13	Octadecanoic acid
93	24.31	0.12	Diazoxon
94	24.52	0.21	Octadecane, 1-bromo-
95	24.80	0.28	Henicosanal
96	24.96	0.70	Silane, ethoxytriethyl-
97	25.29	0.36	N'-(2,4,6(1H,3H,5H)-Trioxopyrimidin-5-ylidenemethyl)-2-nitrobe nzhydrazide
98	25.49	0.29	4,6-Androstadien-17.beta.-ol-3-one
99	25.57	0.34	11-Tetradecyn-1-ol acetate
100	25.85	0.77	4,4'-Dimethoxy-biphenyl-2-carboxylic acid, methyl ester
101	26.04	0.39	1-Chloroeicosane
102	26.17	0.26	Phenanthrene, 7-ethenyl-1,2,3,4,4a,4b,5,6,7,9,10,10a-dodecahydro-1,1,4a,7-tetra methyl-, [4aS-(4a.alpha.,4b.beta.,7.beta.,10a.beta.)]-
103	26.33	0.27	5-Chlorovaleric acid, octadecyl ester
104	26.48	0.17	Pregna-4,6-diene-3,20-dione
105	26.67	0.16	9-Octadecenamide, (Z)-
106	26.77	0.21	Nonadecane
107	26.88	0.31	2,6,10,14,18-Pentamethyl-2,6,10,14,18-eicosapentaene
108	27.00	0.17	1-Selena-2-sila-5-boracyclopent-3-ene, 4,5-diethyl-2,2-dimethyl-3-(1-methylethenyl)-
109	27.08	0.21	Phosphorin, 2,4,6-triphenyl-
110	27.49	0.42	Docosane
111	27.59	0.15	5-formyl-2,3,3',4'-tetramethoxystilbene
112	27.71	0.41	9H-Xanthen-9-one, 1-hydroxy-3,5,6-trimethoxy-

113	27.89	0.12	o-Tolylsulfanyl-acetic acid (4-fluoro-benzylidene)-hydrazide
114	27.97	0.21	Phosphorin, 2,4,6-triphenyl-
115	28.10	0.20	1-Quinoxalineacetic acid, 1,2-dihydro-2-oxo-3-phenyl-, methyl ester
116	28.20	0.11	(1R,2S,8As)-8-oxo-1-carboxymethyl-1,2,5,5-tetramethyl-trans-decalin
117	28.28	0.16	Tricosane
118	28.42	0.18	Sulindac methyl derivative
119	28.50	0.19	(1R,2S,8As)-8-oxo-1-carboxymethyl-1,2,5,5-tetramethyl-trans-decalin
120	28.65	0.10	6-Azaestra-1,3,5(10),8(14)-tetraen-17-one, 3-methoxy-6-methyl-
121	28.71	0.17	(1R,2S,8As)-8-oxo-1-carboxymethyl-1,2,5,5-tetramethyl-trans-decalin
122	28.84	0.12	(1R,2S,8As)-8-oxo-1-carboxymethyl-1,2,5,5-tetramethyl-trans-decalin
123	28.96	0.18	2-Butenenitrile, 2-chloro-3-(4-methoxyphenyl)-
124	29.10	0.12	(1R,2S,8As)-8-oxo-1-carboxymethyl-1,2,5,5-tetramethyl-trans-decalin
125	29.20	0.08	2-Butenenitrile, 2-chloro-3-(4-methoxyphenyl)-
126	29.31	0.35	Stigmastan-3,5-diene
127	29.79	0.17	(1R,2S,8As)-8-oxo-1-carboxymethyl-1,2,5,5-tetramethyl-trans-decalin
128	30.03	0.19	Androsta-1,4-diene-3,11,17-trione
129	30.18	0.14	Tricosanoic acid
130	30.35	0.05	2H-3,9a-Methano-1-benzoxepin, octahydro-2,2,5a,9-tetramethyl-, [3R-(3.alpha.,5a.alpha.,9.alpha.,9a.alpha.)]-
131	30.46	0.13	1-Oxa-2-sila-5-boracyclopent-3-ene, 4,5-diethyl-2,2-dimethyl-3-(1-methylethenyl)-
132	30.67	0.11	2-Butenenitrile, 2-chloro-3-(4-methoxyphenyl)-
133	30.80	0.14	Campesterol
134	31.33	0.10	6-Amino-5-cyano-4-(5-cyano-2,4-dimethyl-1H-pyrrol-3-yl)-2-methyl-4H-pyran-3-carboxylic acid ethyl ester
135	31.53	0.06	1-Benzazirene-1-carboxylic acid, 2,2,5a-trimethyl-1a-[3-oxo-1-butenyl] perhydro-, methyl ester
136	31.66	0.11	2-Bromo-4,5-dimethoxycinnamic acid
137	31.96	0.38	.beta.-Sitosterol
138	32.31	0.08	1-Benzazirene-1-carboxylic acid, 2,2,5a-trimethyl-1a-[3-oxo-1-butenyl] perhydro-, methyl ester
139	32.51	0.04	2-Bromo-4,5-dimethoxycinnamic acid
140	32.64	0.06	3-Piperonyl-2-pyrazolin-5-one
141	32.74	0.10	Thiocarbamic acid, N,N-dimethyl, S-1,3-diphenyl-2-butenyl ester
142	33.03	0.02	1-Benzazirene-1-carboxylic acid, 2,2,5a-trimethyl-1a-[3-oxo-1-butenyl] perhydro-, methyl ester
143	33.17	0.05	2-Bromo-4,5-dimethoxycinnamic acid

144	33.31	0.10	2-Bromo-4,5-dimethoxycinnamic acid
145	33.81	0.07	2-Bromo-4,5-dimethoxycinnamic acid
146	33.95	0.03	2-Bromo-4,5-dimethoxycinnamic acid
147	34.50	0.03	2-(Acetoxymethyl)-3-(methoxycarbonyl)biphenylene
148	34.77	0.05	2-(Acetoxymethyl)-3-(methoxycarbonyl)biphenylene
149	35.17	0.04	2-(Acetoxymethyl)-3-(methoxycarbonyl)biphenylene
150	35.72	0.01	2-(Acetoxymethyl)-3-(methoxycarbonyl)biphenylene
